# PCNA ubiquitylation ensures timely completion of unperturbed DNA replication in fission yeast

**DOI:** 10.1371/journal.pgen.1006789

**Published:** 2017-05-08

**Authors:** Yasukazu Daigaku, Thomas J. Etheridge, Yuka Nakazawa, Mayumi Nakayama, Adam T. Watson, Izumi Miyabe, Tomoo Ogi, Mark A. Osborne, Antony M. Carr

**Affiliations:** 1 Genome Damage and Stability Centre, School of Life Sciences, University of Sussex, Falmer, United Kingdom; 2 Frontier Research Institute for Interdisciplinary Sciences, Tohoku University, Sendai, Japan; 3 Graduate School of Life Sciences, Tohoku University, Sendai, Japan; 4 Research Institute of Environmental Medicine, Nagoya University, Nagoya, Japan; 5 Department of Genome Repair, Atomic Bomb Disease Institute, Nagasaki University, Nagasaki, Japan; 6 Department of Chemistry, School of Life Sciences, University of Sussex, Falmer, United Kingdom; Columbia University, UNITED STATES

## Abstract

PCNA ubiquitylation on lysine 164 is required for DNA damage tolerance. In many organisms PCNA is also ubiquitylated in unchallenged S phase but the significance of this has not been established. Using *Schizosaccharomyces pombe*, we demonstrate that lysine 164 ubiquitylation of PCNA contributes to efficient DNA replication in the absence of DNA damage. Loss of PCNA ubiquitylation manifests most strongly at late replicating regions and increases the frequency of replication gaps. We show that PCNA ubiquitylation increases the proportion of chromatin associated PCNA and the co-immunoprecipitation of Polymerase δ with PCNA during unperturbed replication and propose that ubiquitylation acts to prolong the chromatin association of these replication proteins to allow the efficient completion of Okazaki fragment synthesis by mediating gap filling.

## Author summary

PCNA is a homotrimeric complex that clamps around the DNA to provide a sliding platform for DNA polymerases and other replication and repair enzymes. The covalent modification of PCNA by ubiquitin on lysine reside 164 has been extensively studied in the context of DNA repair: it is required to mediate the bypass of damaged template bases during DNA replication. Previous work has shown that PCNA is modified by ubiquitin during normal S phase in the absence of DNA damage, but the significance of this modification has not been explored. Here we show that, in addition to regulating bypass of damaged bases, lysine 164 ubiquitylation plays a role in ensuring the completion of unperturbed DNA replication.

## Introduction

It is well established that the replication machinery encounters a variety of obstacles and is thus designed with a degree of flexibility. This plasticity of DNA replication depends on both alternative components and regulation by post-translational modification. For example, while genetic and physical studies indicate that the leading and lagging strands are primarily replicated by DNA polymerase ε (Polε) and DNA polymerase δ (Polδ), respectively [1–4], this assignment is flexible: Polδ synthesises the leading strands on rare occasions [5–7], synthesises both strands during viral replication [8] and can sustain cell viability in the absence of Polε [9].

Key to orchestrating enzymes for DNA replication is PCNA, which serves as a scaffold for recruiting many of the numerous enzymes involved, including the replicative DNA polymerases. In addition, PCNA ubiquitylation on lysine164 regulates DNA damage tolerance (DTT). When replication is blocked by damaged DNA bases the Rad6-Rad18 E2-E3 ligase complex binds to single stranded DNA coated with RPA and mono-ubiquitylates PCNA to promote translesion DNA synthesises by non-canonical polymerases [10, 11]. Subsequent to mono-ubiquitylation, PCNA can be poly-ubiquitylated by the Ubc13-Mms2-Rad5 complex [12, 13] to initiate damage bypass by HR-dependent template switching [14]. The level and duration of PCNA ubiquitylation is additionally regulated by constitutive deubiquitylation [15–17].

The prevailing view is that PCNA ubiquitylation is a DNA damage-induced phenomena. This is consistent with the budding yeast situation, where PCNA ubiquitylation is barely detectable in unperturbed S phase but robustly induced in response to replication-blocking DNA lesions [10, 12]. However, PCNA is robustly ubiquitylated during unperturbed replication in fission yeast [18] and significant levels of PCNA ubiquitylation are evident during unperturbed replication in frog extracts and metazoan cells [19, 20]. Several observations suggest that PCNA ubiquitylation is linked to DNA replication: PCNA ubiquitylation is upregulated in response to an increase in canonical replication intermediates [21–23] and a recent synthetic genetic array analysis in budding yeast showed that the PCNA ubiquitylation pathway is genetically correlated with the mechanism of lagging strand DNA synthesis [24]. Moreover, in vitro reconstitution of PCNA ubiquitylation demonstrates that efficient mono-ubiquitylation is coupled to DNA synthesis by Polδ [25].

Despite the accumulating evidence that PCNA ubiquitylation is linked to the processes of DNA replication, there have been no reports that examine if the process of unperturbed DNA replication is influenced by the ubiquitylation of PCNA and the role of this modification during unperturbed S phase remain unclear. To address this question experimentally, we investigated how replication dynamics are influenced by PCNA ubiquitylation in fission yeast. We find that, in the absence of PCNA ubiquitylation DNA replication is slower and that there is an increase in single stranded DNA gaps in S phase cells. We also observe that PCNA ubiquitylation increases the amount of chromatin associated PCNA and influences the recruitment of Polymerase δ. We propose that PCNA ubiquitylation facilitates the completion of Okazaki fragment synthesis.

## Results

**Fig 1:**
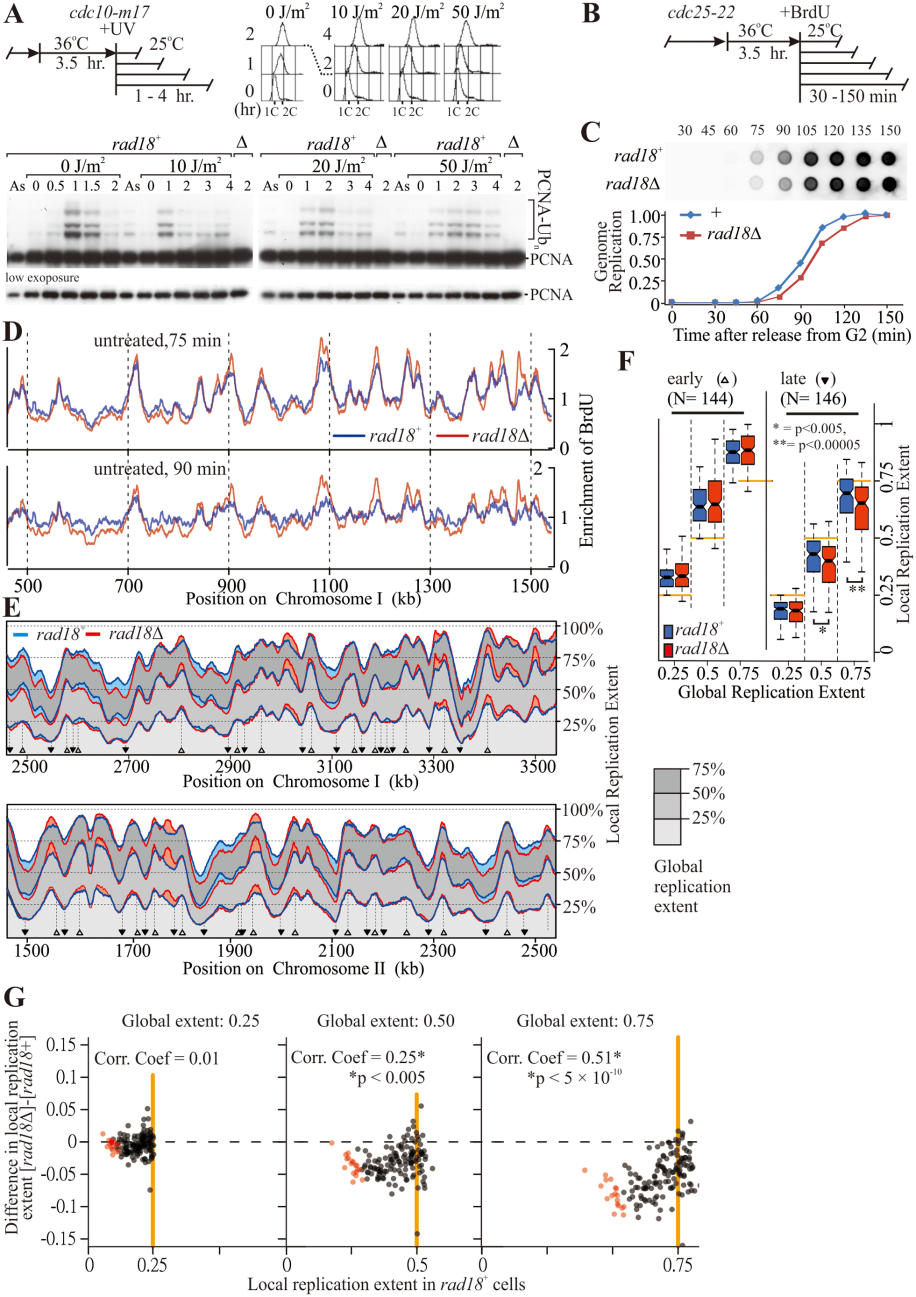
The impact of PCNA ubiquitylation on genome replication. (**A**) Time-course of PCNA ubiquitylation during S-phase in fission yeast cells. Top-left, experimental scheme: cells were synchronised at the G1/S boundary, UV-irradiated, released from the arrest by incubation at 25°C and samples were analysed at the indicated time points for cell-cycle profile (top-right) and PCNA ubiquitylation status (bottom). As = asynchronous cells. (**B**) Experimental scheme to analyse replication dynamics by BrdU incorporation: fission yeast cells are synchronised in G2 and BrdU added to the media when cells were released. Samples are taken at 75 and 90 minutes for the experiments in panel D and at the indicated times for experiments in panels C and E. (**C**) Global BrdU incorporation during synchronous S-phase. Replication was analysed by detecting BrdU in purified total genomic DNA by dot-blot. (**D**) A representative region showing BrdU enrichment following immunoprecipitation and high throughput sequence analysis at two time points for *rad18*+ and *rad18*Δ cells progressing through S phase. (**E**) Replication profiles throughout S phase. Local replication extents were determined following BrdU immunoprecipitation and high throughput sequence analysis. The progression of local replication for each 300 bp chromosomal region was plotted for three conditions: when global genomic replication progression was either 25%, 50% or 75% complete. The blue line represents *rad18*^*+*^, the red line *rad18*Δ. Filled shaded area between these lines highlight the regions where replication extents differ between the two strains; light blue—*rad18*^+^ > *rad18*Δ, light red—*rad18*^+^ < *rad18*Δ. (**F**) Ensemble analysis of replication timing at origins. The distribution of local replication progress when global genomic replication progress is either 25%, 50% or 75% complete. Left: early replicating regions (origins, indicated by open triangle in E). Right: late-replicating regions distal to the origins (indicated by inverted solid triangle in E). (**G**) Correlation between late replication and relative change in replication timing between *rad18*^+^ and *rad18*Δ. The latest replicating regions in *rad18Δ* cells are shown in pink (see S4 Fig).

In fission yeast PCNA is ubiquitylated during unperturbed S phase [18] and is not significantly further induced by UV-induced DNA damage during S-phase (Fig 1A and S1A and S1B Fig). Cells arrested in early S phase by hydroxyurea maintained high levels of PCNA ubiquitylation (S1C Fig) and after irradiation in S phase PCNA ubiquitylation persisted for longer (Fig 1A), likely due to the slowed S phase progression. Thus, the PCNA ubiquitylation promoted by UV-irradiation of asynchronous fission yeast cultures (S1D Fig) is primarily a consequence of cells accumulating in S phase. To explore what replication defects result in PCNA ubiquitylation we examined PCNA-Ub in selected temperature-sensitive replication mutants. We observed that inactivation of enzymes required for lagging strand synthesis (DNA ligase 1, Polδ), but not enzymes associated with replisome progression (the MCM complex, Polε), resulted in elevated ubiquitylation levels at lysine 164 of PCNA (S1E and S1F Fig). Collectively, these results indicate that the accumulation of lagging strand intermediates [21–23], but not fork stalling *per se*, are a major cause of PCNA ubiquitylation.

### PCNA ubiquitylation contributes to the progression of replication forks

If incomplete lagging strand synthesis activates PCNA ubiquitylation, it is possible that PCNA-Ub participates in the completion of Okazaki fragment synthesis. To examine this possibility, we first determined the contribution of PCNA ubiquitylation to the progression of unperturbed S phase by assessing replication dynamics in synchronised populations (Fig 1B–1F). Since *S*. *pombe* Pcn1 can be modified on lysine 164 by either ubiquitin or SUMO, we first examined cells defective for the Rhp18 E3-ligase (Rhp18 is the *S*. *pombe* homolog of *S*. *cerevisiae* Rad18. For clarity, we refer to this E3 ligase as Rad18 through the text). While S phase entry was slightly delayed in *rad18*Δ cells (Fig 1C), bulk replication progression proceeded with similar kinetics when assessed by total bromodeoxyuridine (BrdU) accumulation (Fig 1C). In contrast, while cells carrying the mutation of the ubiquitylated PCNA residue, *pcn1-K164R*, also slightly delayed S phase entry, their progress through S phase was also defective (S2A and S2B Fig). Importantly, *rad18*Δ was epistatic with *pcn1-K164R* for the slight delay to S phase entry (S2A Fig), confirming that the delay seen in *rad18*Δ cells is PCNA ubiquitylation dependent. It is unclear why the *pcn1-K164R* mutation also conferred a ubiquitylation-independent defect in S phase progression (S2A Fig). We observed that replication timing was also perturbed and that Polε DNA association during S phase was reduced (see below) in a manner that was independent of the Pli1 SUMO ligase. As *pcn1-K164R* is thus clearly acting as a hypomorphic allele, we concentrated our analysis on the *rad18* deletion mutant cells.

To establish if PCNA ubiquitylation affected the DNA replication kinetics of specific loci we examined enrichment of BrdU across the genome during mid to late S phase by BrdU-IP in *rad18*^+^ and *rad18*Δ cells (Fig 1D). This showed changes to the replication dynamics, with advanced replication close to origins and delayed replication for the inter-origin regions. Because relative BrdU enrichment between two samples does not directly reflect relative replication kinetics (the two samples will not be at exactly the same point in S phase), we performed independent replication time courses for *rad18*^+^ and *rad18*Δ cells and normalised for replication progression in order to directly compare DNA replication timing across the genome (Fig 1E, see Materials and methods for details). Replication progression was calculated at each local region of the genome when the global genome replication level was either 25, 50 or 75%. I.e. we used the global extent of replication to standardise comparisons between *rad18*^+^ and *rad18*Δ strains such that the extent of local replication was compared between strains with equivalent global levels. *rad18*Δ cells showed delayed replication at regions distal to replication origins which are, relative to origins, late replicating (light blue, Fig 1E). This was compensated for by higher local replication at many origin-associated regions that are relatively early replicating (light red, Fig 1E). Some additional peaks were also observed, for example regions 1770-kb region in Chr. II and 3320-kb in Chr. III, suggesting reduced fork progression rates are partially compensated for by firing cryptic origins [26]. The distribution of BrdU at genomic regions surrounding origins would be expected to become wider as S phase progressed (ultimately it would be flat at the end of S phase). Consistent with our hypothesis that replication fork progression is subtly delayed in *rad18*Δ cells (Fig 1E), we observed that deletion of *rad18* resulted in a narrower distribution of BrdU later in S phase when compared to *rad18*^+^ control cells (S3A–S3E Fig). Control experiments where we allowed cells to progress into S phase in the presence of hydroxyurea confirmed that the two strains initiated S phase at the same origins and confirm that our sequencing methodology is reproducible (S3B Fig).

To examine further whether *rad18*Δ caused delayed replication in regions that replicate late, a meta-analysis was performed by computationally identifying replication origins and analysing the relatively late replicating inter-origin regions. As shown in Fig 1F the local replication extent of the early replicating origins was not perturbed in *rad18*Δ. In contrast, later replicating regions show a significant decrease in their extent of replication, even when adjusted for the global replication amounts. This effect was particularly striking in the regions that were amongst the last to be replicated (Fig 1G). Analysis of the specific loci that were most under-replicated in *rad18*Δ cells (S4A and S4B Fig) showed they correspond to those loci that we previously demonstrated to be the last to be replicated in wild type cells [5]. These data demonstrate that the lack of PCNA ubiquitylation delays replication fork progression, with the cumulative effect manifesting most obviously at late replicating regions.

### DNA-loaded PCNA is stabilised by ubiquitylation

**Fig 2:**
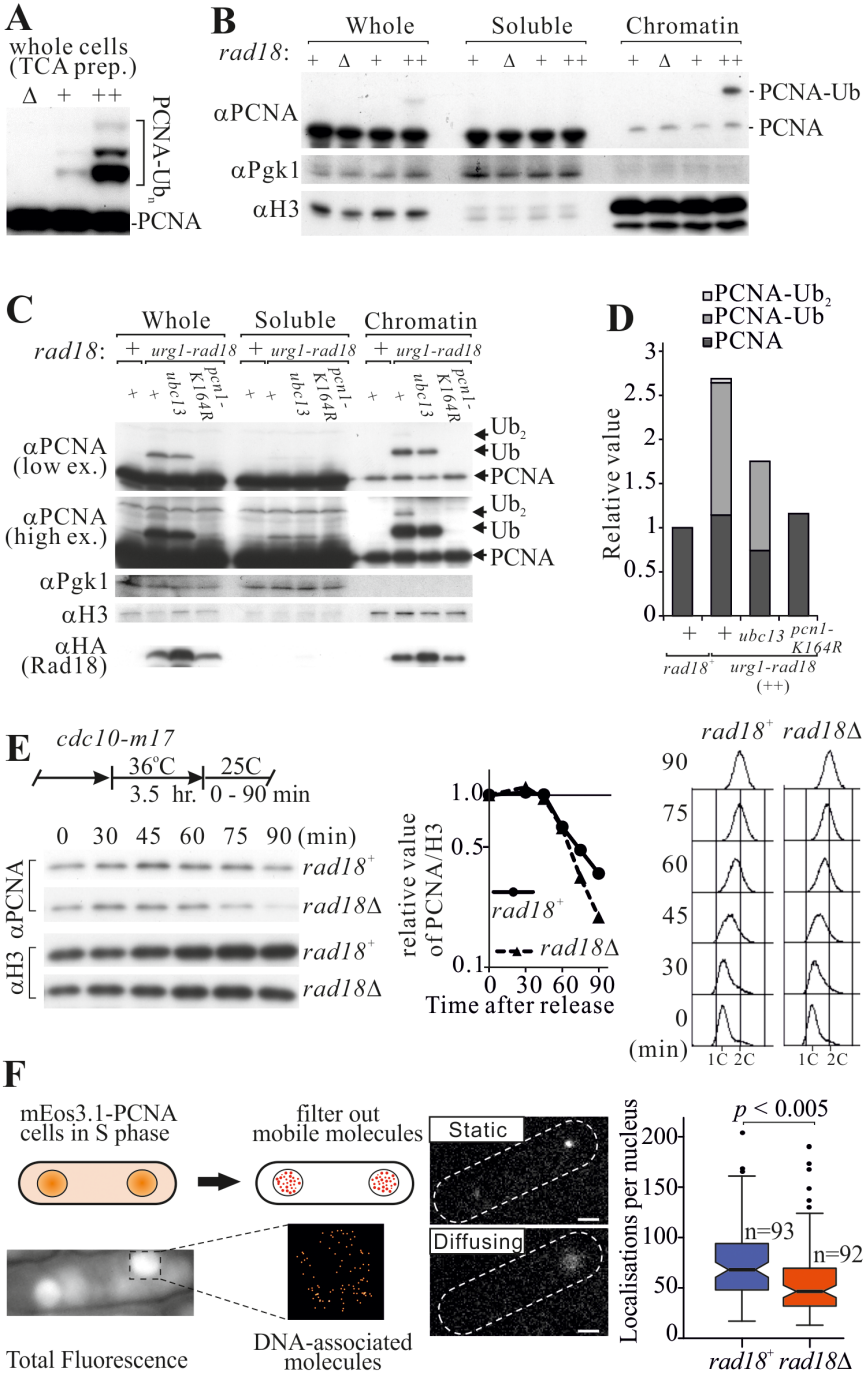
PCNA ubiquitylation influences PCNA chromatin association. **(A**) PCNA ubiquitylation levels in log phase cells when Rad18 is absent (Δ), expressed normally (+) or upregulated in P_urg1_-*rad18* cells (++). Ubiquitylation was assessed following protein extraction in denaturing condition (TCA prep). (**B**) PCNA chromatin association. The cells shown in panel A expressing different levels of Rad18 and thus harbouring distinct level of PCNA ubiquitylation were analysed for PCNA chromatin association. Pgk1 (a soluble cytosolic protein) and histone H3 were used as controls. (**C**) Dependency of chromatin association on PCNA-K164 and Ubc13. An equivalent experiment as shown in panel B for *pcn1-K164R* and *ubc13* mutant backgrounds. (**D**) Quantification of the modified and unmodified PCNA from C. (**E**) Chromatin association of PCNA during S phase progression. Top left, experimental scheme: cells were synchronised at the G1/S boundary by temperature shift and PCNA chromatin association (bottom left panel) and cell cycle profiles (right) monitored at the indicated time points after release into S phase. The relative amounts of chromatin-associated PCNA were quantified (centre) (**F**) Quantification of DNA-associated PCNA by single molecule PALM imaging. Top left: schematic showing how motion blurring filters out mobile molecules. Bottom left: example of the visualisation of only the DNA-associated molecules. Middle: example of static localised molecule (top) and the typical signal from a diffusing molecule (bottom). Right: box plots of the numbers of visualised immobile PCNA molecules in the S phase nuclei of the indicated strains. Bi-nucleate (S phase) *mEos3-pcn1* cells (expressing mEos3-tagged PCNA) were imaged and mEos3-PCNA localisations quantified per nucleus.

PCNA is loaded during DNA replication, functions as the replicative clamp and remains chromatin associated until the polymerase has finished replication and ligation is complete. We speculated that PCNA ubiquitylation may contribute to PCNA retention on the chromatin. However, in native cell extracts PCNA is progressively deubiquitylated, compromising the ability to measure PCNA ubiquitylation during chromatin association assays. To overcome this limitation, we increased the level of PCNA ubiquitylation by engineering a strain, P_urg1_-*rad18*, where *rad18*^+^ is under the control of an inducible promoter (Fig 2A). Fractionation of cell extracts following *rad18* induction revealed that ubiquitylated PCNA was preferentially associated with chromatin (Fig 2B) in a manner dependent on K164 ubiquitylation (Fig 2C and 2D). This suggests the modification contributes to the stability of PCNA chromatin association. Consistent with this, shut-off of *rad18*^+^ transcription from P_urg1_ when combined with induced Rad18 degradation resulted in rapid PCNA disassociation from chromatin, concomitant with deubiquitylation (S5 Fig). Because it is not practical to assay native fission yeast extracts for endogenous levels of ubiquitylated PCNA on chromatin due to its deubiquitylation by isopeptidase in native extracts we compared the total chromatin-associated PCNA in *rad18*^+^ and *rad18*Δ cells during S phase. In *rad18*^+^ cells, PCNA accumulated in S phase and gradually diminished towards the completion of replication. Comparatively, in *rad18*Δ cells, the amount of chromatin associated PCNA decreased during the late stages of replication (Fig 2E). This is reminiscent of the predominant effect of loss of PCNA ubiquitylation manifesting at late replicating regions (Fig 1F and 1G).

We verified the observed effect of Rad18 loss on PCNA chromatin association using a photo-activated localization microscopy (PALM)-based technique that directly visualises DNA-associated PCNA [27]. Briefly, this method exploits motion blurring to selectively eliminate signals arising from rapidly diffusing molecules, allowing visualisation of low mobility signals derived from DNA-associated molecules (Fig 2F). Previously we reported that low mobility PCNA (mEos3.1-Pcn1) is notably enriched during S phase [27]. Deletion of *rad18* significantly reduced the fraction of these molecules (Fig 2F, right), thus confirming that PCNA-K164 ubiquitylation results in increased amounts of loaded PCNA during unperturbed S phase.

### Polδ association with PCNA is increased by PCNA ubiquitylation

**Fig 3:**
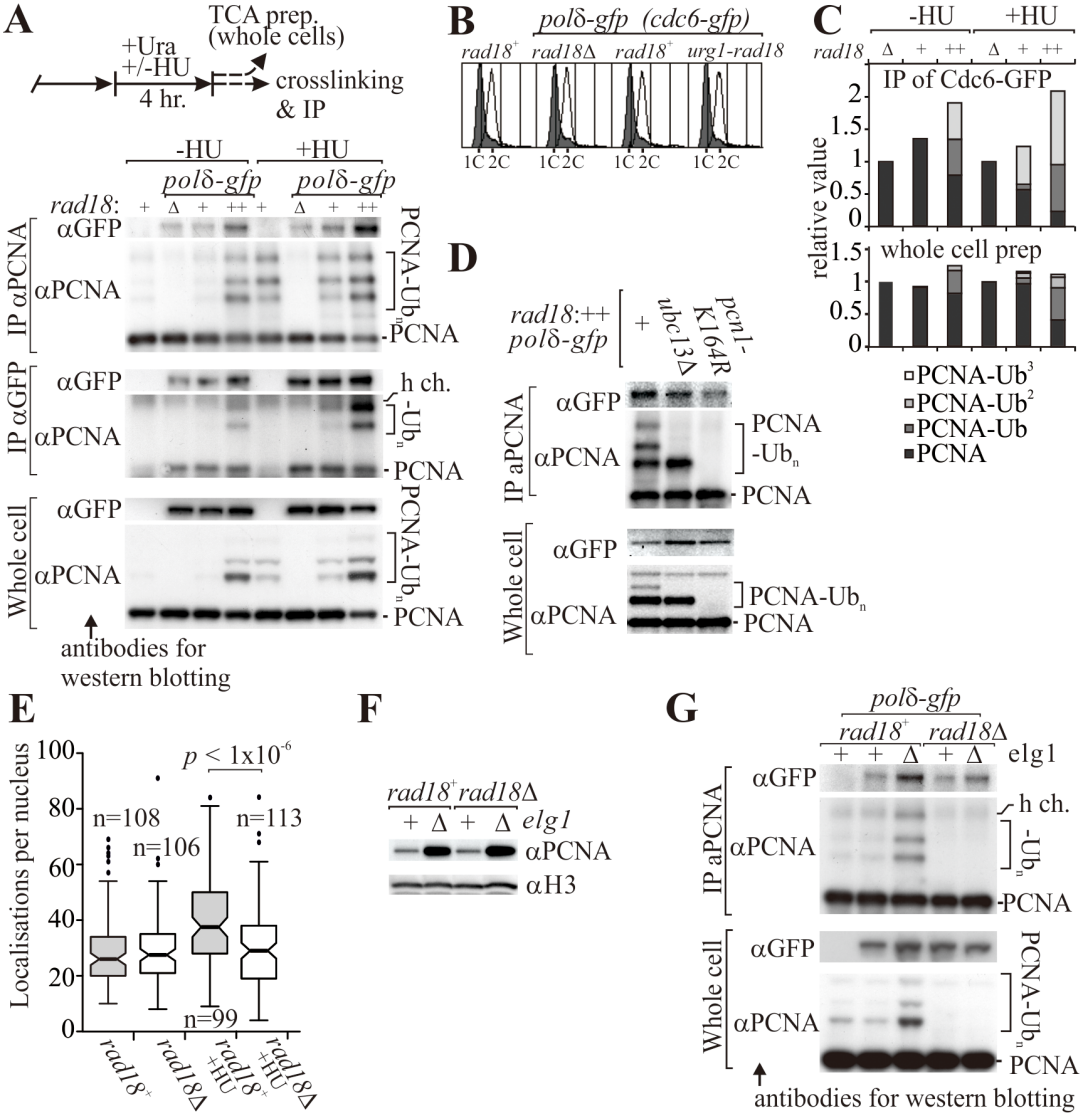
PCNA ubiquitylation contributes to the interaction of Polδ with the clamp. (**A**) Assessing co-immunoprecipitation of Polδ and PCNA. Top: experimental scheme for co-immunoprecipitation experiments. Cells were either treated, or not, with hydroxyurea (HU) and TCA extracts prepared to monitor PCNA modification (whole cell) or soluble extracts prepared for immunoprecipitation with either anti-PCNA or anti-GFP (the catalytic subunit of Polδ is GFP tagged). Bottom: comparison of the Polδ - PCNA interaction in *rad18*Δ (Δ), wild-type (+) or *urg1-rad18* cells (++) cells that exhibiting distinct levels of PCNA ubiquitylation (see Fig 2A). (**B**) Cell-cycle profiles. Open histograms -HU, grey histograms +HU. (**C**) Quantification of modified forms of PCNA that was co-immunoprecipitated with Polδ (“IP with αGFP” in a) and in whole cell extracts. (**D**) Co-immunoprecipitation of Polδ in P_urg1_-*rad18* cells is partially dependent on *ubc13*. (**E**) Quantification of DNA-associated Polδ by single molecule PALM imaging (see Fig 2F for details). (**F**). Increased chromatin association of PCNA in the *elg1*Δ genetic background. Chromatin was fractionated from the indicated strains and probed for Pcn1 and a histone H3. (**G**) Co-immunoprecipitation of Polδ with PCNA in *elg1*^+^ (+) and *elg1*Δ (Δ) cells in the *rad18*^+^ and *rad18*Δ genetic backgrounds.

One possible explanation for the increased amount of chromatin-associated PCNA accompanying K164 ubiquitylation is that this contributes to the function of DNA polymerases during DNA replication. In unchallenged cells we could detect the association of Polδ, but not Polε, with PCNA by immunoprecipitation (S6A and S6B Fig). This would be consistent with the higher PCNA-dependency of Polδ function [28–30], but may equally reflect the lower levels of DNA-associated Polε during S phase when compared to Polδ. Increased PCNA ubiquitylation (by Rad18 overexpression via P_urg1_-*rad18*) increased Polδ co-immunoprecipitation with anti-PCNA without influencing cell cycle profiles (Fig 3A and 3B). PCNA ubiquitylation and co-immunoprecipitation were also both enhanced by hydroxyurea treatment of *rad18*^+^ and P_urg1_-*rad18* cells. Thus, Polδ: PCNA co-immunoprecipitation intensity scaled with PCNA ubiquitylation (Fig 3A and 3B). We also noted that the PCNA which co-immunoprecipitated with Polδ was biased toward ubiquitylated forms (Fig 3C) and that the loss of poly-ubiquitylation (*ubc13* deletion) showed an intermediate decrease in co-immunoprecipitation of Polδ when compared to loss of all ubiquitylation (*pcn1-K164R*) (Fig 3D). Using the PALM motion blurring assay (see Fig 2F) we did not detect a decrease in the Polδ immobile fraction in untreated S phase *rad18*Δ cells (Fig 3E), possibly because our assay is insufficiently sensitive. However, when *rad18*Δ cells were arrested within S phase by hydroxyurea treatment, the fraction of low mobility Polδ molecules decreased when compared to *rad18*^+^ controls, providing support for the contention that PCNA ubiquitylation contributes to Polδ function.

PCNA recruits DNA polymerases after it is loaded [31] and the affinity of PCNA: Polδ binding is not influenced by K164 ubiquitylation [32]. Thus, the increased Polδ-PCNA association could be accounted for purely by the increased amount of PCNA on DNA due to ubiquitylation inhibiting clamp unloading. This predicts that increasing PCNA chromatin association independently of its ubiquitylation status would lead to increased Polδ: PCNA co-immunoprecipitation. To address this, we examined Polδ-PCNA association in cells deleted for *elg1*, where PCNA chromatin association is enhanced due to inactivation of the Elg1 unloader (Fig 3F) [33]. Loss of Elg1 resulted in an increase in Polδ co-immunoprecipitation with PCNA in both *rad18*^+^ and *rad18*Δ backgrounds (Fig 3G). This result demonstrated that the amount of loaded PCNA relates to the level of PCNA-polymerase association, although we cannot rule out the possibility that additional factors that directly respond to PCNA ubiquitylation can also influence the association.

PCNA ubiquitylation is proposed to help ‘replace’ replicative polymerases with non-canonical polymerases. We therefore examined co-immunoprecipitation of several DNA damage tolerant polymerases, Polη, Polκ and Polζ, with PCNA (S6C Fig). Marginal Polη: PCNA co-immunoprecipitation was observed in P_urg1_-*rad18* cells, where PCNA ubiquitylation levels were high, consistent with the ubiquitin-binding zinc-finger domain of Polη directing PCNA association. Co-immunoprecipitation of Polκ or Polζ with PCNA was not detectable, presumably due to sparse protein levels (S6C Fig). Taken together, these data indicated that the non-canonical polymerases do not appreciably outcompete Polδ for association with ubiquitylated PCNA. Consistent with this, neither of Polη, Polκ nor Polζ were responsible for the altered BrdU incorporation observed in *rad18*Δ cells during unperturbed S phase (S6D Fig).

**Fig 4:**
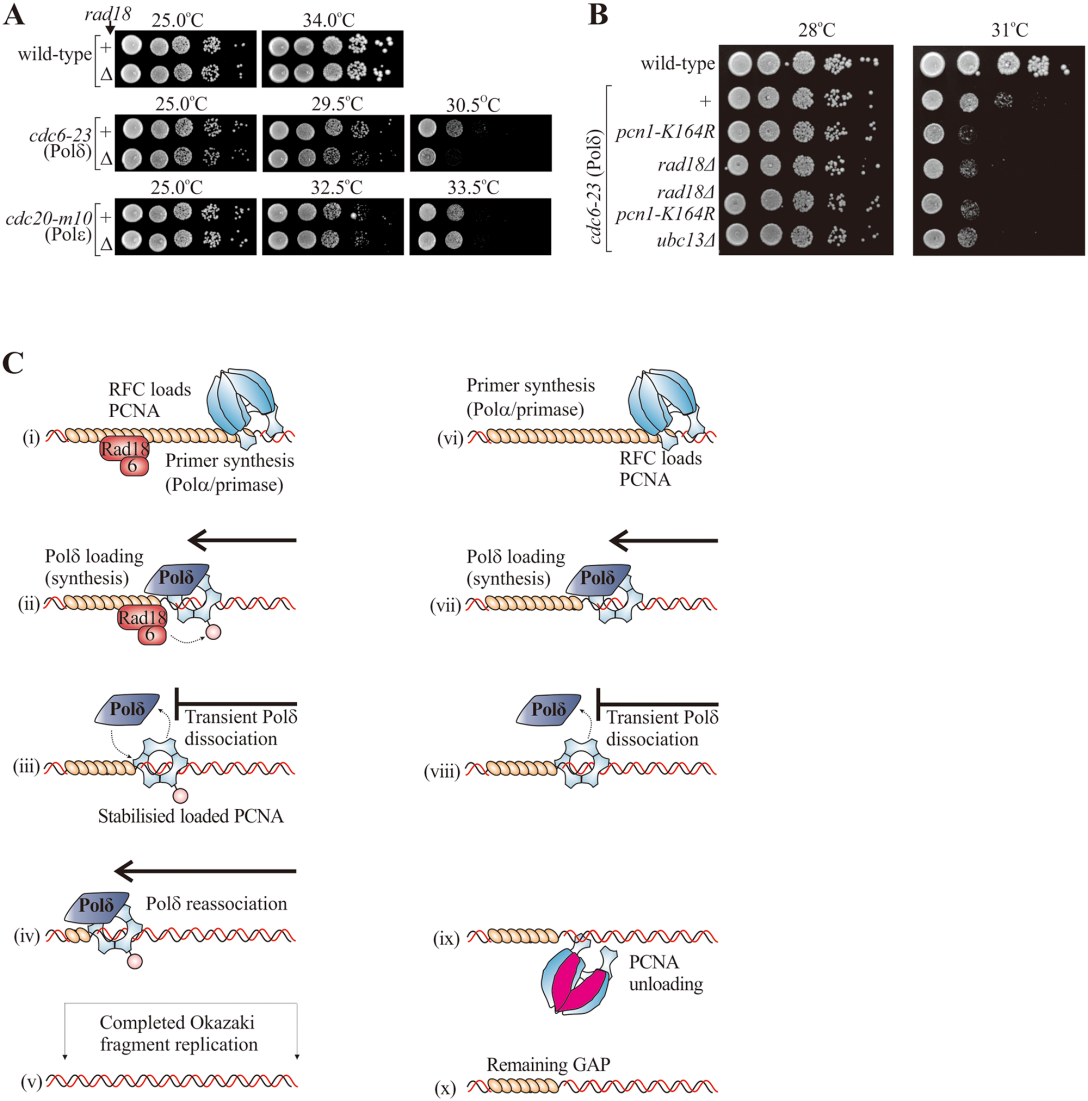
PCNA ubiquitylation predominantly affects Polδ function. **(A**) Spot tests showing the influence of PCNA ubiquitylation on cell growth/viability in genetic backgrounds compromised for Polε or Polδ function. *cdc20-m10* and *cdc6-23* are temperature sensitive mutations in the catalytic subunits of Polε and Polδ, respectively. (**B**) The effect of *pcn1-K164R* mutation and *rad18* deletion on the temperature sensitivity of *cdc6-23* (Polδ) are epistatic. (**C**) Model. Left: Schematic of Okazaki fragment synthesis. (i) Following priming by Polα-primase PCNA is loaded by RFC. (ii) Polδ associates with PCNA and synthesis begins. Rad18-Rad6 mono-ubiquitylates PCNA. (iii) Stochastic dissociation of Polδ from PCNA. Ubiquitylation prevents PCNA dissociation/unloading. (iv) Polδ re-associates with PCNA and synthesis resumes. (v) Okazaki fragment synthesis is completed. Right, a potential effect of loss of Rad18: (vi) Following priming by Polα-primaae PCNA is loaded by RFC. (vii) Polδ associates with PCNA and synthesis begins. (viii) Stochastic dissociation of Polδ from PCNA. (ix) PCNA is not ubiquitylated and thus can dissociate (possibly unloaded by Elg1). (x) Okazaki fragment synthesis is not completed and a gap remains.

To establish if PCNA modification influences Polδ and Polε function we examined synthetic genetic interactions between *rad18*Δ and temperature sensitive (ts) polymerase mutations (Fig 4A). For *cdc6-23*, (Polδ-ts), concomitant *rad18*Δ reduced the restrictive temperature, consistent with PCNA ubiquitylation enhancing Polδ activity. Importantly, this synthetic genetic interaction was also observed for *pcn1-K164R* and combining both *rad18*Δ and *pcn1-K164R* showed no additive effect (Fig 4B). For *cdc20-m10* (Polε-ts) *rad18*Δ did not affect the restrictive temperature, suggesting Polε activity is not influenced by PCNA ubiquitylation. Consistent with this, when we examined the fraction of low mobility Polε in S phase cells using PALM motion blurring, we did not detect a significant change in when *rad18* was deleted (S6E Fig). Interestingly, when we examined Polε mobility in a *pcn1-K164R* background, a significantly lower fraction of Polε displayed low mobility in S phase cells (S6E Fig). This phenomenon was not observed in a *pli1* deletion mutant (S6F Fig). Thus, the K164R mutation has effects beyond that of PCNA ubiquitylation (c.f. S2 Fig) which are unlikely to be related to modification by small Ub-like molecules.

### PCNA ubiquitylation during unperturbed replication promotes gap filling

During DDT ubiquitylation of PCNA promotes ssDNA gap filling opposite DNA lesions [22, 34, 35]. We have confirmed (S1E Fig) that PCNA ubiquitylation is induced following dysfunction of Okazaki fragment synthesis and demonstrated that this increases the fraction of Polδ co-immunoprecipitating with PCNA (Fig 3A–3C) and can contribute to the chromatin association of this lagging strand polymerase (Fig 3E). Since Polδ repeatedly disassociates from and re-associates with the template during synthesis [32], relatively long lived ssDNA gaps may occur stochastically between Okazaki fragments. We reasoned that PCNA ubiquitylation could act to supress or repair such events via a DTT-like gap filling mechanism during unperturbed S-phase (Fig 4C). This predicts that the absence of PCNA ubiquitylation would result in ssDNA gap accumulation during DNA replication.

**Fig 5:**
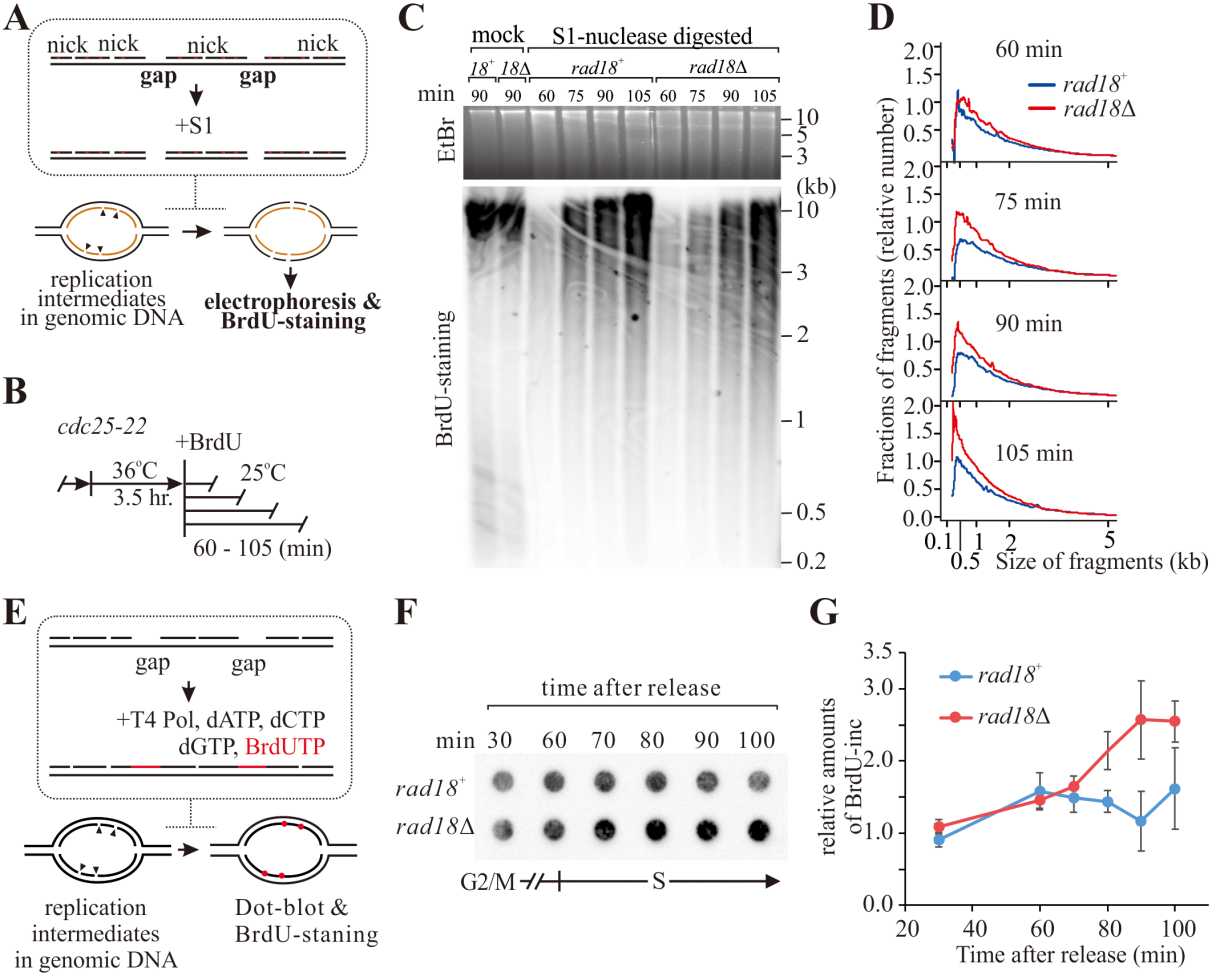
PCNA ubiquitylation mediates repair of ssDNA gaps during S-phase. (**A**) Schematic of the S1 nuclease assay [36] used to monitor gaps during DNA replication. (**B**) Experimental scheme. *cdc25-22* cells were synchronised at G2 phase by temperature shift then released into the cell cycle and samples harvested at the indicated time points. (**C**) Analysis of S1-nuclease digested total DNA. Samples were subjected to agarose gel electrophoresis and visualised by ethidium bromide (EtBr) staining to reveal the total DNA (top) and subjected to BrdU antibody detection to reveal newly synthesised strands (bottom). (**D**) Quantification of S1-nuclese digested fragments. Fractions of fragments along the axis of fragment length were calculated from the intensity of BrdU signals in C. (See S7 Fig for the calculation steps). (**E**) Schematic of the BrdU assay used to monitor gaps during DNA replication. (**F**) detection of BrdU incorporated into the indicated DNA samples prepared in agarose plugs. Experimental scheme as in B. (**G**) Quantification of BrdU incorporation. Plotted data are derived from three independent experiments.

To estimate the extent of ssDNA gaps *in vivo*, we first utilised an S1 nuclease-based assay [36] previously developed for detecting ssDNA in replicated molecules (Fig 5A). By calculating the distribution of DNA fragment sizes from gel intensities (S7 Fig) we infer that *rad18*Δ cells displayed increased DNA fragmentation when compared to *rad18*^+^ cells, with small (< 1 kb) fragments accumulating in *rad18*Δ throughout S phase (Fig 5B–5D). As an alternative assay, we BrdUTP labelled ssDNA gaps in genomic DNA prepared in agarose plugs. When DNA from r*ad18*Δ cells was compared to *rad18*^+^, increased signal was evident in mid to late S phase (Fig 5E–5G). These two experiments support a model where PCNA ubiquitylation occurs between Okazaki fragments (Fig 4C) and prevents the accumulation of ssDNA gap during unperturbed S phase (see Discussion).

## Discussion

Here we have used the fission yeast model to demonstrate that, in addition to its known role in DNA damage tolerance, PCNA K164-ubiquitylation contributes to the timely completion of unperturbed DNA replication. Our results show that PCNA association with chromatin is stabilised by PCNA-K164 ubiquitylation during S phase. We also observed an increased co-immunoprecipitation of Polδ with PCNA when PCNA is ubiquitylated and we provide evidence that the chromatin association of Polδ is promoted by PCNA ubiquitylation. In budding yeast, PCNA ubiquitylation is barely detectable in unperturbed S phase [22] and robustly induced in response to DNA lesions that block the canonical replicative DNA polymerases [10, 12]. Consequently, PCNA ubiquitylation has been studied almost exclusively in the context of its key role in DNA damage tolerance [37]. In contrast, in fission yeast PCNA is robustly ubiquitylated during unperturbed S phase [18] and this is not significantly further induced if DNA is damaged during S phase. Budding and fission yeast thus represent opposite ends of what appears to be a spectrum. We note that both yeasts have approximately similar genome sizes and there is no evidence to suggest that fission yeast suffers from elevated levels of spontaneous DNA damage. Interestingly, mammalian cells exhibit both S phase-dependent PCNA ubiquitylation and DNA damage induced PCNA ubiquitylation (see S8A and S8B Fig).

It is currently not known what underlies the differences between organisms in terms of PCNA ubiquitylation in unperturbed S phase. However, as Rad18 is activated by regions of single stranded DNA it is possible that PCNA ubiquitylation is reflecting the extent of ssDNA present when DNA replication is active. In support of this, in budding yeast a defect in short-flap Okazaki fragment processing caused by compromising the function of the Fen1 flap endonuclease, which normally processes the 5’end of Okazaki fragments, induced detectable levels of PCNA ubiquitylation [24]. This is explained by the accumulation of long 5’ ssDNA flaps that bind RPA and activate Rad18 ubiquitylation. However, when Okazaki fragment-processing is proficient, the vast majority of flap structures are cleaved by Fen1 when they are 1 or 2 nucleotide in length [38]. Thus, Okazaki fragment processing is unlikely to be a significant source of ssDNA during unperturbed S phase. We have shown here that the lack of PCNA ubiquitylation leads the accumulation of ssDNA gaps during S phase in fission yeast. We propose that the dynamics of Polδ disassociation from PCNA result in stochastic formation of transient gaps during lagging strand synthesis. These gaps trigger Rad18-dependent ubiquitylation of PCNA, which stabilises PCNA on the DNA, allowing association of Polδ and rapid gap resolution. In the absence of PCNA ubiquitylation, a proportion of these gaps persist and thus gaps are detected in our assays. The generation of transient gaps during lagging stand synthesis likely explains the fact that PCNA is ubiquitylated during S phase in this organism.

In support of fission yeast generating increased regions of ssDNA during unperturbed DNA replication (when compared to budding yeast) we note that the abrogation of recombination pathways in fission yeast (e.g. *rad51*Δ or r*ad52*Δ mutants) causes a much more severe growth defect than the equivalent loss of recombination pathways in budding yeast and that the combination of *rad51* deletion with *rad18* or *pcnl-K164R* results in synthetic lethality (S9 Fig). This suggests that homologous recombination and DTT pathways cooperatively repair ssDNA gaps, which may be abundant compared to *S*. *cerevisiae*.

In considering the origin of ssDNA during S phase that we observe in *S*. *pombe* and the differential PCNA ubiquitylation between *S*. *pombe* and *S*. *cerevisiae* in unperturbed S phase, it is interesting to consider that the kinetics of Polδ holo-enzyme dissociation from PCNA. It has recently been reported [32] that the *S*. *cerevisiae* enzyme is more processive than its human counterpart: human Polδ dissociates more rapidly from PCNA than its budding yeast counterpart and it was estimated that ~14–31% of human Okazaki fragments are completed by two independent Polδ: PCNA association events. Conversely, >99% of budding yeast Okazaki fragments are predicted to be completed by a single Polδ: PCNA interaction. While the kinetics of *S*. *pombe* Polδ dissociation from PCNA has not been studied, the fact that PCNA ubiquitylation is strongly influenced by the intermediates of lagging strand DNA synthesis [21–23] (see S1E and S1F Fig) is consistent with the fission yeast PCNA ubiquitylation pathway, in addition to regulating translesion synthesis during DTT, functioning to maintain accurate Okazaki fragment synthesis in the face of frequent Polδ: PCNA dissociation.

Okazaki fragment synthesis is necessarily coupled, either directly or indirectly, to the movement of replication forks. Approximately 10^5^ and 10^7^ Okazaki fragments are synthesised per cell cycle in fission yeast cells and human cells, respectively. The potential for failure during this process as a consequence of premature Polδ dissociation would therefore need to be minimised by ensuring the re-association of Polδ and completion of Okazaki fragment synthesis. We propose that this is facilitated by PCNA ubiquitylation, which ensures that PCNA is not prematurely unloaded. The fact that we show that the loss of PCNA ubiquitylation results in the accumulation of ssDNA gaps during unperturbed S phase in *S*. *pombe* (Fig 5) supports our model. Intriguingly, preliminary analysis (S10 Fig) showed the positive effect of PCNA ubiquitylation on PCNA chromatin association is evident only when the Elg1 unloader complex is active, suggesting that PCNA ubiquitylation may inhibit its unloading by Elg1, a PCNA unloading factor currently characterised only in in *S. cerevisiae* [33].

In *S*. *cerevisiae* yeast, SUMOylated PCNA is the predominant modification during unperturbed S phase [12, 39]. Previous work showed that Elg1 preferentially interacts with SUMO-modified PCNA [40]. However, Elg1 unloads both unmodified and SUMOylated forms of PCNA, an event which in budding yeast requires the ligation of Okazaki fragments [41]. However, in fission yeast, and in human cells, SUMOylated PCNA is much harder to detect and the SUMO-interacting motifs identified in *S*. *cerevisiae* Elg1 are not conserved. Thus, the influence of PCNA SUMOylation is unlikely to be prominent and we propose that the effect of PCNA ubiquitylation on stabilising PCNA is more predominant in fission yeast cells and potentially higher eukaryotes. It has also been suggested that the unloading of PCNA in response to HU or MMS in *S*. *cerevisiae* is dependent on its ubiquitylation and concomitant activation of the DNA damage checkpoint [42]. One interpretation of this apparent contradiction could be that, under extensive replication stress, checkpoint activation changes the response to PCNA ubiquitylation. Alternatively, this may again reflect a difference between the two organisms in the regulation of PCNA unloading.

In fission yeast a significant proportion of PCNA is ubiquitylated during unperturbed S phase. To avoid a global engagement of error prone DNA polymerases, we propose that the replicative polymerases remain the preferred binding partners for ubiquitylated PCNA. However, when a replicative polymerase is stalled at a blocking lesion, the ubiquitin binding domain-containing polymerases are provided an increased opportunity to sample the damaged base. In budding yeast the situation is distinct: PCNA is not significantly ubiquitylated in unperturbed S phase, but is robustly ubiquitylated in response to a replicative polymerase arrested at a lesion. Thus, we would predict that the binding kinetics for the replicative and error-prone DNA polymerases will be different between the two organisms in order to maintain the same biological outcomes: an appropriate balance between unsuitable use of error prone DNA polymerases during unperturbed S phase (to minimise constitutive mutagenesis) and their appropriate use during DNA damage tolerance to maximise cell survival in response to DNA damage [43].

In summary, our analysis shows that PCNA ubiquitylation, in addition to controlling DNA damage tolerance pathway usage, also participates in the timely completion of unperturbed DNA synthesis. We propose that this function is related to the increased association of ubiquitylated PCNA with chromatin. We suggest that, when Polδ stochastically dissociates during Okazaki fragment synthesis, the consequent ssDNA results in PCNA ubiquitylation which ensures it remains DNA-associated to facilitate the recapture of Polδ and completion of Okazaki fragment synthesis.

## Materials and methods

### Yeast strains and molecular genetics

Standard *S*. *pombe* genetic and molecular techniques were employed as described previously [44]. The BrdU-incorporating strains have been already reported [45]. Polδ-GFP cells were constructed by introducing the sequence encoding GFP into the N-terminal of the *cdc6* gene on *S*. *pombe* genome based on the Cre-loxP method [46]. Polε-GFP cells were constructed by introducing GFP at the C-terminal of *cdc20* gene using PCR-based integration [47]. *P*_*urg1*_*-rad18* strains were based on *rad18*Δ cells in which ORF of the *rad18* gene fused with the AID degron construct [48] was used to replace the endogenous *urg1* ORF [49].

### Cell cycle synchronisation in human cells

U2OS cells were cultured in Dulbecco’s modified Eagle’s medium supplemented with 10% foetal bovine serum (DMEM-FBS10%) in a 5% CO2 atmosphere. The medium was exchanged with one containing 400 ng/ml of nocodazol. Following 18 hr incubation, mitotic cells were detached by gentle shaking of the culture vessel and passaged in DMEM-FBS10%. Cells were then either UV-irradiated (254 nm peak; 20J/m2), or not, 2 hr prior to sampling. At the indicated time points cells were sampled and then subjected to immunoblotting with anti-PCNA antibody (mouse monoclonal, PC10 clone, Abcam). To determine the S-phase fraction of the synchronised cells, 5μM if EdU was added into an aliquot and EdU positive cells scored 2 hr after EdU addition [50]. 1BR3hTERT cells were cultured in DMEM-FBS10% and the medium was exchanged with DMEM without FBS. Following 15 days, cells were passaged into DMEM-FBS10%. Cells were UV-irradiated and scored for S-phase fraction as described. Cell lines from GDSC collection. Authenticated 2015 by STR profiling.

### BrdU-labelling assay

*cdc25-22* cells harbouring the constructs for BrdU-incorporation were grown to exponential phase (0.2 x10^6^ /ml) at 25°C and synchronised at G_2_ phase by incubation at 36°C for 3.5 hr. After adding bromodeoxyuridine (0.5 μM), cells were further incubated at 25°C. At relevant time points, 1x10^8^ cells were pelleted and subjected to genomic DNA extraction. To detect total BrdU incorporation, dot blotting was performed as previously described [34]. The intensity of BrdU-incorporation was established by quantifying the signal using an ImageQuant LAS 4000 imager (GE Healthcare Life Sciences). Global replication rates for each time point after release from G2 phase were estimated by dividing signal intensities at each time-points by that for 150 min, at which genome replication was completed. Local replication rates were established from BrdU-IP-Sequencing.

### Analysis of BrdU-IP sequencing

Paired-end reads from high throughput sequencing were aligned to the *S*. *pombe* genome sequence (ASM294v2.23: chromosomes I, II and III, downloaded from 'PomBase’ website) using bowtie2–2.2.2. From the alignment data the position of the centre of each read was calculated and the number of reads in 300bp-bins across genome counted. The Perl program converting alignment data to count data: ‘sam-to-count.pl’ is available on the GitHub website (https://github.com/yasukasu/sam-to-bincount). The counts at the chromosome coordinate x, C_B_(t, x)–the BrdU-IP sample derived from cells at the t-min time point, C_I_(0, x)–the input sample derived from cells before release from G2 (t = 0), were normalised with the total number of reads: N_B_(t, x) = C_B_(t, x)/ΣC_B_(t, x), N_I_(t, x) = C_I_(t, x)/ΣC_I_(t, x). Enrichments for BrdU-incorporated fragments were calculated: E(t, x) = N_B_(t, x)/N_I_(t, x). As BrdU is an analogue of thymine and its enrichment is thus likely to be biased towards A/T rich regions, the dataset of enrichment was normalised using the A/T-ratio of each 300-bp bin AT(x): E’(t, x) = E(t, x)/AT(x). Moving average of E’(t, x) with 8 bins at both side were calculated and plotted (Fig 1D). To estimate the extent of local replication, enrichments across the genome were multiplied by the global replication amount G(t) determined from the dot-blot assay (Fig 1C): L(t,x) = E’(t, x) ×G(t). These were then normalised with that of the last time point, at which all the cells had completed genome replication: L’(t,x) = L(t,x)/L(160, x). To obtain a function of local replication extent, data of multiple time points at each 300 bp (L’(70, x), L’(75, x), L’(80, x), L’(85, x), L’(90, x)) were fitted with a cumulative normal distribution function in which the global replication amount is variable, F(G, x). Using this function, Local replication extent when the global replication was 25%, 50% or 75% completed was determined: F(0.25, x), F(0.5, x) and F(0.75, x). Fig 1E–1G and S4 Fig is derived from these datasets. The custom R scripts used for this computational analysis are available on request.

### Chromatin isolation

Whole cell extracts were prepared by spheroplast lysis using Zymolyase 100T (Seikagaku) and lysing enzyme (Sigma-Aldrich). Extracts were fractionated into soluble and chromatin-bound fractions by centrifugation through a sucrose cushion [51].

### Immunoprecipitation

5 x 10^8^ exponentially growing cells in 50 ml YE medium were treated with 1% formaldehyde for 15 min at RT under agitation. The crosslinking reaction was quenched by adding 2.5 ml of 2.5 M glycine. Cells were washed with ice-cooled PBS, pelleted and re-suspended in 700 μl pf RIPA buffer (50mM HEPES pH7.5, 1mM EDTA, 140 mM NaCl, 1% Triton X-100, 0.1% (w/v) sodium deoxycholate) supplemented with complete protease inhibitor (Roche), 1 mM AEBSF & 1μg/ml pepstatin (Sigma-Aldrich). After adding zirconia/silica beads (biospec), cells were ribolysed (6 bursts of 30 sec at speed 6.5 in a FastPrep ribolyser (MP-Biomedicals). 300μl of cell lysate was sonicated (7 cycles; 30 sec on, 30 sec off) using a bioruptor pico (diagenode), l μl of benzonase (novagen) added and incubated for 20 min on ice. The lysate was then centrifuged (14000 rpm for 30 min in a microfuge) and the supernatant transferred to new tube. 30 μl was kept as the ‘input’ sample. 2 μg of anti-GFP antibody (rabbit IgG, A11122, Life technologies) or anti-PCNA antibody [18] was added to the isolated cell extract. After a 3 hr incubation at 4°C with gentle agitation, 20 μl of magnetic G protein dynabeads (Life technologies) was added and incubated for a further 1 hr. Beads were washed twice with RIPA buffer and once with TE. Following addition of 60 μl of elution buffer, beads were incubated at 65°C for 15 min. Supernatant was isolated as the ‘IP’ sample. Laemmli buffer was added into both ‘IP’ and ‘input’ samples and western blots were interrogated with anti-PCNA or anti-GFP (mouse IgG clones 7.1 and 13.1, Roche).

### S1-endonuclease assay

1 x 10^8^ cells were incubated in YE media containing 50 μg/ml of BrdU and subjected to genomic DNA extraction [44]. 2 μg of extracted DNA was digested with 1 μl of S1-nuclease (Life Technologies) using the manufactures buffer in a 20 μl reaction mixture. The reaction was stopped by the addition 2 μl of 0.5 M EDTA and heating to 70°C for 10 min. The complete reaction mixture was subjected to agarose (1.5%) electrophoresis. DNA was transferred onto GeneScreen Plus membrane (PerkinElemer) by neutral capillary transfer and the BrdU signal detected by the immunoblotting [34]. Normalisation of the BrdU incorporation intensities to the fraction of S1-digested fragments was performed as previously described for alkaline digested DNA [52].

### BrdU gap-filling assay

4 x 10^7^ cells were harvested and subjected to Zymolyase 100T (0.5 mg/ml, Seikagaku) and lysing enzyme (1mg/ml, Sigma-Aldrich) treatment in 1ml of spheroplasting buffer (20 mM citrate-phosphate buffer, 50 mM EDTA and 1.2M sorbitol). After spheroplasting, cells were re-suspended in 80 μl of spheroplasting buffer without enzymes, mixed with 80 μl of 2% agarose (SeaPlaque GTG agarose) and then 20 μl volume of agarose plugs were prepared. Plugs were washed with detergents and treated with Protease K as described previously [53]. Three plugs were subjected to treatment with T4 polymerase (6 units, New England Biolabs) and dNTP with BrdUTP (Sigma-Aldrich) instead of dTTP (200 μM each) in 100 μl at 37°C overnight. DNA was recovered from plugs by phenol/chloroform extraction and applied to dot blots (Scie-Plas Ltd.). BrdU signal was detected as described above.

### PALM microscopy

Analysis of DNA binding *in vivo* by PALM was performed as previously described using a custom-built microscope system [27]. Photoconversion and excitation of mEos3 molecules was controlled by continuous wave illumination with 405nm and 561nm laser light. The intensity of the 405nm laser was modulated during the imaging such that the number of photoconverted molecules for any one frame was kept low to reduce the chances of overlapping static molecules, or the possibility of blurring molecules masking static localisations. Laser intensities at the sample were calculated as 0.1-1W/cm^2^ (405nm) and 1kW/cm^2^ (561nm). Camera EM gain was set at 250 and exposure time for each frame was 350ms. Typical data acquisition consisted of 3000–4000 frames and 6000–10000 frames for polymerases and PCNA respectively. Data sets were built from of a minimum of 3 biological repeats. Raw image data were processed using a custom ImageJ 2D-Gaussian fitting routine as previously described^23^. Code available on GitHub: https://github.com/aherbert/GDSC-SMLM and as a Fiji update site (GDSC SMLM). Scale bar 1.5 micometers.

### Accession numbers

Data files for BrdU-IP sequence have been deposited in the Gene Expression Omnibus database under accession number GSE70033.

## Supporting information

S1 Fig Observation of PCNA ubiquitylation in S phase cells, HU-treated cells and mutants in which lagging strand DNA synthesis is compromised. (**A**). A repeat of the experiment shown in Fig 1A with quantification (right). (**B**) Time-course of PCNA ubiquitylation during S-phase in fission yeast cells. Top-left, experimental scheme: cells were synchronised in G2 phase using *cdc25-22* and released from the arrest by incubation at 25°C. At the onset of S phase (45 min after release), cells were UV-irradiated (T = 0). Samples were analysed at the indicated time points for cell-cycle profile (top-right), ratios of cells during mitosis and septated cells, which is the indicator of being in S-phase (bottom-right) and for PCNA ubiquitylation status (bottom-left). As = asynchronous cells. (**C**) Observation of PCNA ubiquitylation in HU-treated cells. Following releases of *cdc10-m17* cells from the G1/S boundary, cells were incubated with media containing 10mM hydroxyurea. (**D**) PCNA in UV irradiated asynchronous cultures. Exponentially growing cells were irradiated with the indicated dose of UV and then incubated at 30°C for 1–3 hr. (**E**) PCNA ubiquitylation analysed in response to specific defects in DNA replication. The indicated temperature sensitive replication mutants were shifted from the permissive to the restrictive temperature and analysed for cell cycle profile (top) and PCNA ubiquitylation status (bottom). *cdc10* encodes the homolog of E2F subunit (G1 arrest negative control), *cdc20* encodes the catalytic subunit of Polε (leading strand polymerase), *cdc6* encodes the catalytic subunit of Polδ (lagging strand polymerase), *cdc17* encodes DNA ligase I, *cdc21* encodes the Mcm4 homolog (replicative helicase subunit). (**F**) Dependency of induced PCNA ubiquitylation due to dysfunction of Polδ or DNA ligase on Lys164 of PCNA, Rad18, Ubc13 and Mms2. The indicated cells were incubated at 36°C (restrictive temperature) for 4hr.
(TIF)

S2 Fig The effect of *rad18* deletion and p*cn1-K164R* mutation on cell cycle progression and DNA replication. (**A**) BrdU-incorporation into genomic DNA during the subsequent S-phase after G2 arrest and release of *cdc25-22* cells (see Fig 1 for details). (**B**) Fraction of septated cells after release from G2 phase.
(TIFF)

S3 Fig Genome-wide replication profiles in *rad18*^+^ and *rad18*Δ cells. (**A**) Experimental scheme: *cdc25-22* cells were synchronised at G2 and released. At the indicated time points cells were harvested and subjected to BrdU-IP-seq. (**B**) Representative view of BrdU-incorporation at a genomic region. The data for untreated time points is reproduced from Fig 1D to provide a comparison for the HU treated time points. The counts of reads at the chromosome coordinate x (300-bp bin), CB(x)–BrdU-IP sample, CI(x)–input sample derived from cells before release from G2, were normalised with the total number of reads: NB(x) = CB(x)/ΣCB(x), NI(x) = CI(x)/ΣCI(x). Enrichment of BrdU-incorporated fragments was calculated and plotted: E(x) = NB(x)/NI(x). (**C**) The computational detection of replication tracks. Replication origins were detected as peaks (blue dots) in the profiles in HU-treated cells. The regions where more than 15 kb of shoulder was associated both sides of the origin were designated as replication tracks (pink boxes). (**D**) The Overlay of BrdU enrichment data from the ‘untreated, 75min’ sample for the designated replication tracks. Blue: *rad18*^+^, red: *rad18*Δ. Enrichments for each track were normalised with the value at the peak and plotted: i.e. the maximum value of each plotted track is 1. (**E**) Averages of normalised BrdU enrichment data of all replication tracks.
(TIF)

S4 Fig Lack of PCNA ubiquitylation results in a significant delay to DNA replication at the latest replicating latest of the genome. (**A**) Scatter plot of local replication progress at late replicating regions. The sub-population of loci that deviate substantially from the distribution of replication rates in *rad18*Δ cells is boxed. (**B**) The late replicating regions indicated in A are marked (pink lines) on the global replication profile [5] of the three fission yeast chromosomes.
(TIF)

S5 Fig De-ubiquitylation of PCNA results in its release from chromatin. PCNA loaded onto chromatin was monitored upon the shut-off of Rad18. Following growth under inducing conditions of P_urg1_-*rad18-aid* (presence of uracil (+Ura), the expression was shut off by washing out uracil (+Ura to -Ura). Residual auxin-degron tagged Rad18 was degraded (+Auxin) or not (-Auxin) by the addition of auxin.
(TIF)

S6 Fig Co-immunoprecipitation of the replicative polymerases Polε or Polδ with PCNA and the motion blur assay for Polε. (**A**) Whole cell extracts were prepared from untagged and Polδ-gfp tagged (*cdc6-gfp*) cells and immunoprecipitated with anti-GFP antibody following protein-crosslinking. (**B**) Equivalent immunoprecipitation was performed with extracts derived from Polε-gfp tagged (*cdc20-gfp*) cells. (**C**) Co-immunoprecipitation of Polζ, Polη and Polκ using anti-PCNA antibody in cells that exhibit distinct levels of PCNA ubiquitylation (see panel a). * = GFP-tagged polymerase. h ch. = heavy chain. (Bottom right: Alternative exposure of gel emphasising the GFP tagged polymerase bands. (**D**) BrdU incorporation in cells which lack individual translesion polymerase; Polζ (*rev3*Δ), Polη(*eso1-D147N*) or Polκ (*kap1*Δ). Two independent experiments were shown. (**E,F**) DNA-associated Polε was quantified by single molecule PALM imaging (see Fig 2F for details). ns = not significant.
(TIF)

S7 Fig Normalisation of BrdU incorporation intensities to the fraction of S1-digested fragments. Calculations were performed as previously described [52]. (**A**) Fluorescence intensity curves derived from BrdU incorporation in Fig 5C. (**B**) The horizontal axis of the graph in A was converted from the position on the gel (pixel) to size of S1-digested fragment (kb). (**C**) Fluorescent intensity, i.e. the amount of BrdU incorporation per pixel, was converted to the fraction of fragments along with the axis of fragment length.
(TIF)

S8 Fig Analysis of PCNA ubiquitylation in human cells. (**A**) PCNA ubiquitylation during cell cycle progression in human U2OS cells. Top-left, experimental scheme: cells were synchronised with nocodozol, released into fresh media and samples either irradiated, or not irradiated before harvesting at the indicated time points. The fraction of S phase cells was determined by EdU staining (top-right) and PCNA ubiquitylation status was monitored by western blot (bottom). (**B**) Equivalent experiment using 1BR3hTERT immortalized human fibroblasts synchronised by serum starvation.
(TIF)

S9 Fig Synthetic lethality of homologous recombination and DDT pathways. Tetrad analysis of a cross between *rad52*Δ cells and *rad18*Δ or *pcn1-K164R* cells. *rad52*Δ colonies exhibit a slow growth phenotype whereas *rad52*Δ *rad18*Δ or *rad52*Δ *pcn1-K164R* double mutants are lethal.
(TIF)

S10 Fig Quantification of DNA-associated PCNA in *elg1*-deleted cells by single molecule PALM imaging. Motion blur of mEos3-PCNA in S phase cells in *rad18*^+^ and *rad18*Δ cells (see Fig 2F) and in the *rad18*^+^ e*lg1*Δ and *rad18*Δ *elg1*Δ backgrounds.
(TIF)
