# Supplementary figures and images for: PCNA ubiquitylation ensures timely completion of unperturbed DNA replication in fission yeast
Source: PLoS Genet. 2017 May 8;13(5):e1006789. doi: 10.1371/journal.pgen.1006789 (PMC5440044; doi:10.1371/journal.pgen.1006789)

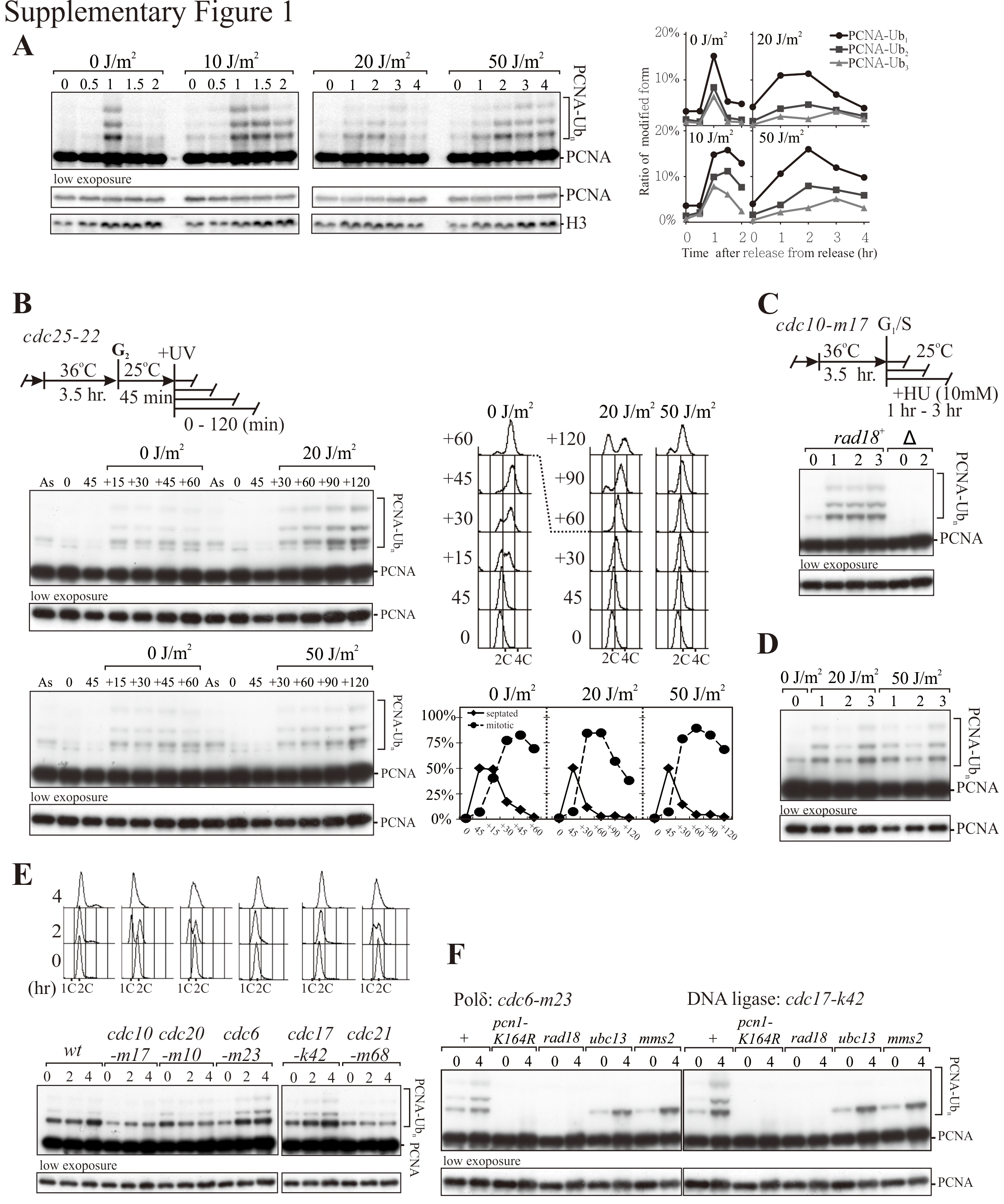

Supplement: S1 Fig — (A). A repeat of the experiment shown in Fig 1A with quantification (right). (B) Time-course of PCNA ubiquitylation during S-phase in fission yeast cells. Top-left, experimental scheme: cells were synchronised in G2 phase using cdc25-22 and released from the arrest by incubation at 25°C. At the onset of S phase (45 min after release), cells were UV-irradiated (T = 0). Samples were analysed at the indicated time points for cell-cycle profile (top-right), ratios of cells during mitosis and septated cells, which is the indicator of being in S-phase (bottom-right) and for PCNA ubiquitylation status (bottom-left). As = asynchronous cells. (C) Observation of PCNA ubiquitylation in HU-treated cells. Following releases of cdc10-m17 cells from the G1/S boundary, cells were incubated with media containing 10mM hydroxyurea. (D) PCNA in UV irradiated asynchronous cultures. Exponentially growing cells were irradiated with the indicated dose of UV and then incubated at 30°C for 1–3 hr. (E) PCNA ubiquitylation analysed in response to specific defects in DNA replication. The indicated temperature sensitive replication mutants were shifted from the permissive to the restrictive temperature and analysed for cell cycle profile (top) and PCNA ubiquitylation status (bottom). cdc10 encodes the homolog of E2F subunit (G1 arrest negative control), cdc20 encodes the catalytic subunit of Polε (leading strand polymerase), cdc6 encodes the catalytic subunit of Polδ (lagging strand polymerase), cdc17 encodes DNA ligase I, cdc21 encodes the Mcm4 homolog (replicative helicase subunit). (F) Dependency of induced PCNA ubiquitylation due to dysfunction of Polδ or DNA ligase on Lys164 of PCNA, Rad18, Ubc13 and Mms2. The indicated cells were incubated at 36°C (restrictive temperature) for 4hr. (TIF) [file pgen.1006789.s001.tif]

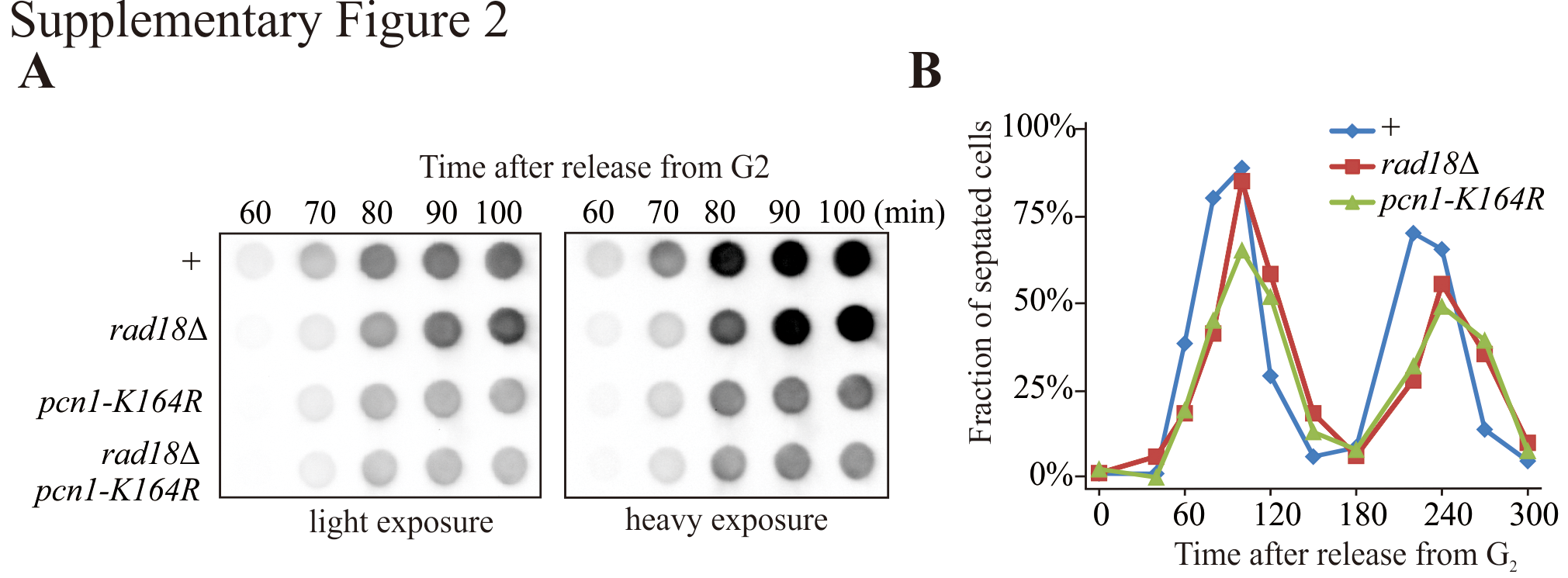

Supplement: S2 Fig — (A) BrdU-incorporation into genomic DNA during the subsequent S-phase after G2 arrest and release of cdc25-22 cells (see Fig 1 for details). (B) Fraction of septated cells after release from G2 phase. (TIFF) [file pgen.1006789.s002.tiff]

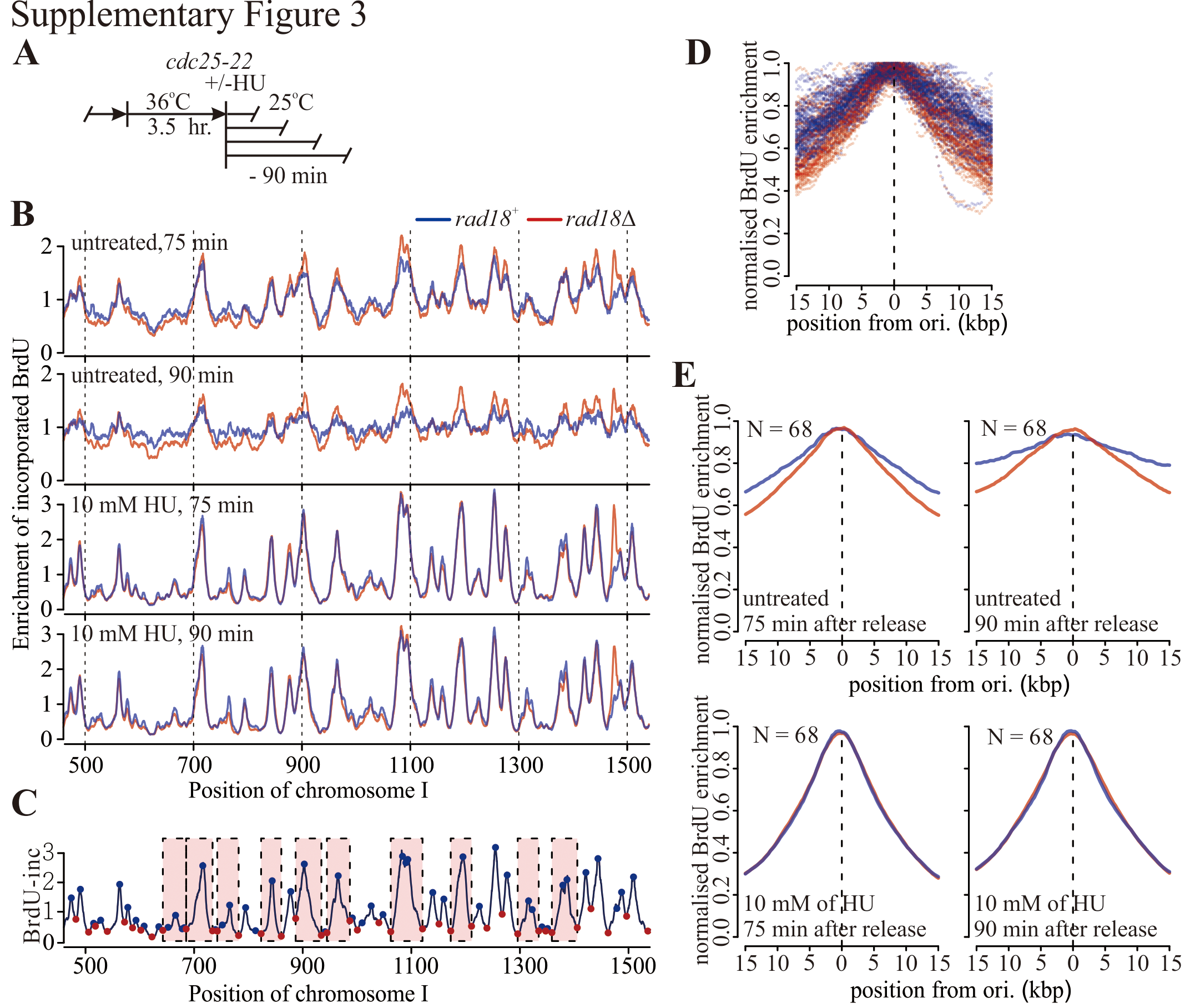

Supplement: S3 Fig — (A) Experimental scheme: cdc25-22 cells were synchronised at G2 and released. At the indicated time points cells were harvested and subjected to BrdU-IP-seq. (B) Representative view of BrdU-incorporation at a genomic region. The data for untreated time points is reproduced from Fig 1D to provide a comparison for the HU treated time points. The counts of reads at the chromosome coordinate x (300-bp bin), CB(x)–BrdU-IP sample, CI(x)–input sample derived from cells before release from G2, were normalised with the total number of reads: NB(x) = CB(x)/ΣCB(x), NI(x) = CI(x)/ΣCI(x). Enrichment of BrdU-incorporated fragments was calculated and plotted: E(x) = NB(x)/NI(x). (C) The computational detection of replication tracks. Replication origins were detected as peaks (blue dots) in the profiles in HU-treated cells. The regions where more than 15 kb of shoulder was associated both sides of the origin were designated as replication tracks (pink boxes). (D) The Overlay of BrdU enrichment data from the ‘untreated, 75min’ sample for the designated replication tracks. Blue: rad18+, red: rad18Δ. Enrichments for each track were normalised with the value at the peak and plotted: i.e. the maximum value of each plotted track is 1. (E) Averages of normalised BrdU enrichment data of all replication tracks. (TIF) [file pgen.1006789.s003.tif]

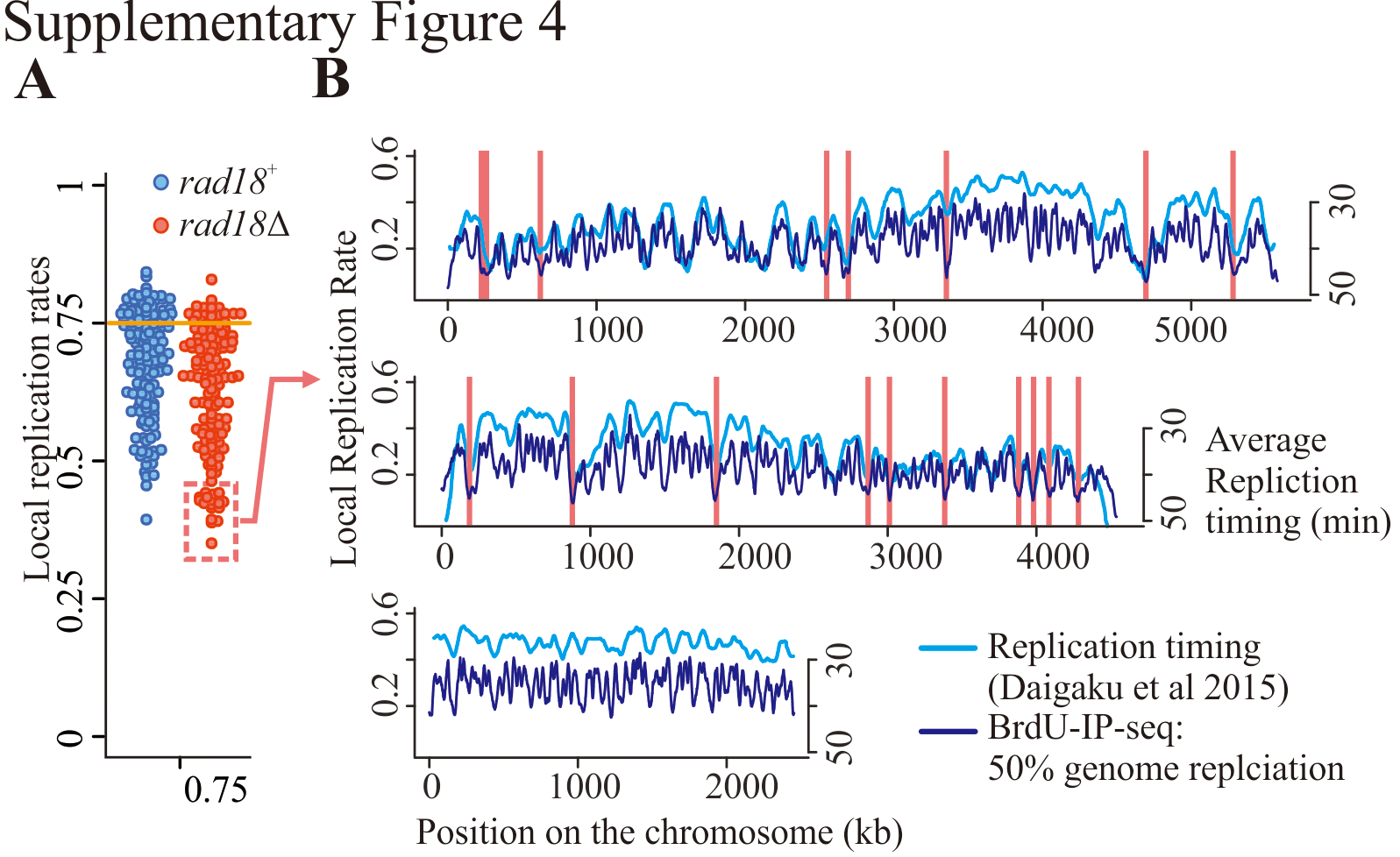

Supplement: S4 Fig — (A) Scatter plot of local replication progress at late replicating regions. The sub-population of loci that deviate substantially from the distribution of replication rates in rad18Δ cells is boxed. (B) The late replicating regions indicated in A are marked (pink lines) on the global replication profile [5] of the three fission yeast chromosomes. (TIF) [file pgen.1006789.s004.tif]

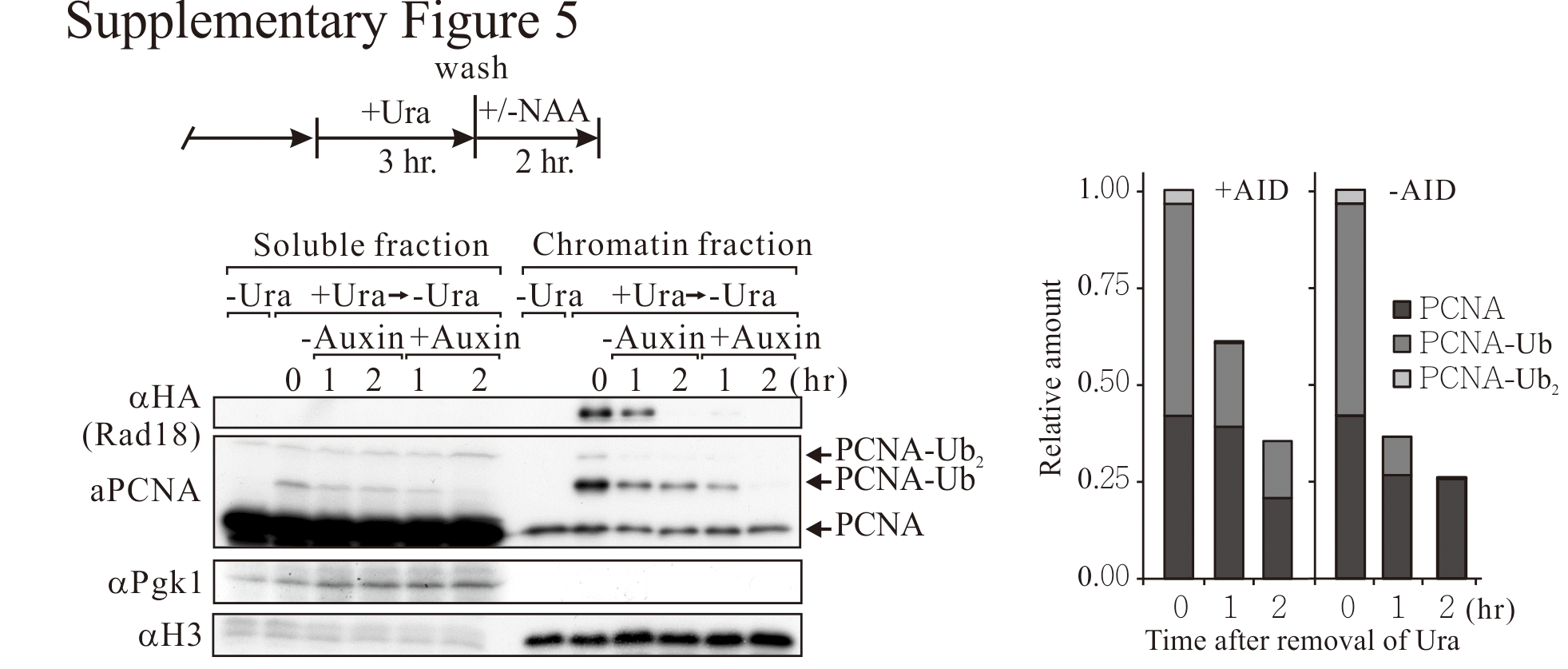

Supplement: S5 Fig — PCNA loaded onto chromatin was monitored upon the shut-off of Rad18. Following growth under inducing conditions of Purg1-rad18-aid (presence of uracil (+Ura), the expression was shut off by washing out uracil (+Ura to -Ura). Residual auxin-degron tagged Rad18 was degraded (+Auxin) or not (-Auxin) by the addition of auxin. (TIF) [file pgen.1006789.s005.tif]

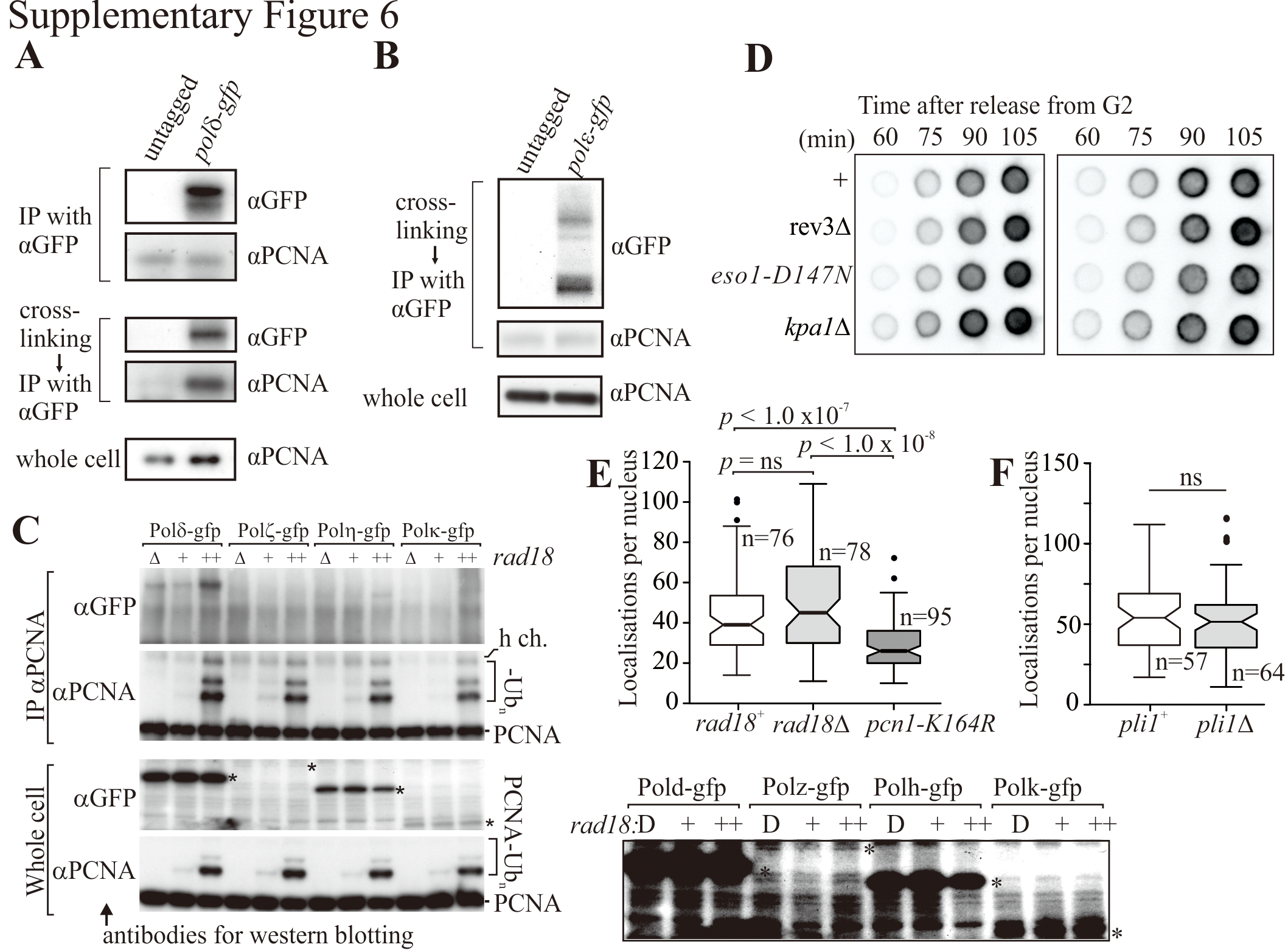

Supplement: S6 Fig — (A) Whole cell extracts were prepared from untagged and Polδ-gfp tagged (cdc6-gfp) cells and immunoprecipitated with anti-GFP antibody following protein-crosslinking. (B) Equivalent immunoprecipitation was performed with extracts derived from Polε-gfp tagged (cdc20-gfp) cells. (C) Co-immunoprecipitation of Polζ, Polη and Polκ using anti-PCNA antibody in cells that exhibit distinct levels of PCNA ubiquitylation (see panel a). * = GFP-tagged polymerase. h ch. = heavy chain. (Bottom right: Alternative exposure of gel emphasising the GFP tagged polymerase bands. (D) BrdU incorporation in cells which lack individual translesion polymerase; Polζ (rev3Δ), Polη(eso1-D147N) or Polκ (kap1Δ). Two independent experiments were shown. (E,F) DNA-associated Polε was quantified by single molecule PALM imaging (see Fig 2F for details). ns = not significant. (TIF) [file pgen.1006789.s006.tif]

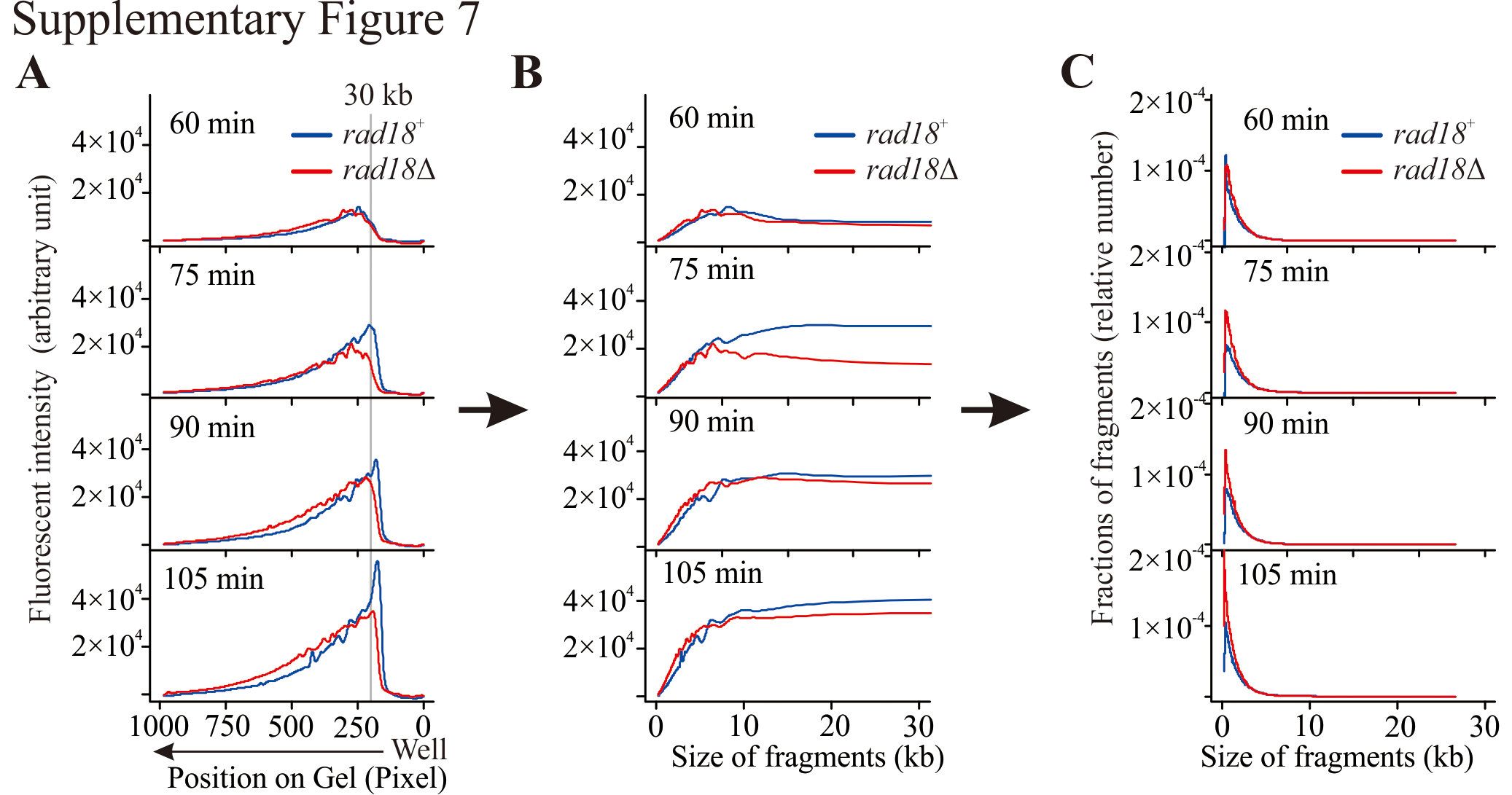

Supplement: S7 Fig — Calculations were performed as previously described [52]. (A) Fluorescence intensity curves derived from BrdU incorporation in Fig 5C. (B) The horizontal axis of the graph in A was converted from the position on the gel (pixel) to size of S1-digested fragment (kb). (C) Fluorescent intensity, i.e. the amount of BrdU incorporation per pixel, was converted to the fraction of fragments along with the axis of fragment length. (TIF) [file pgen.1006789.s007.tif]

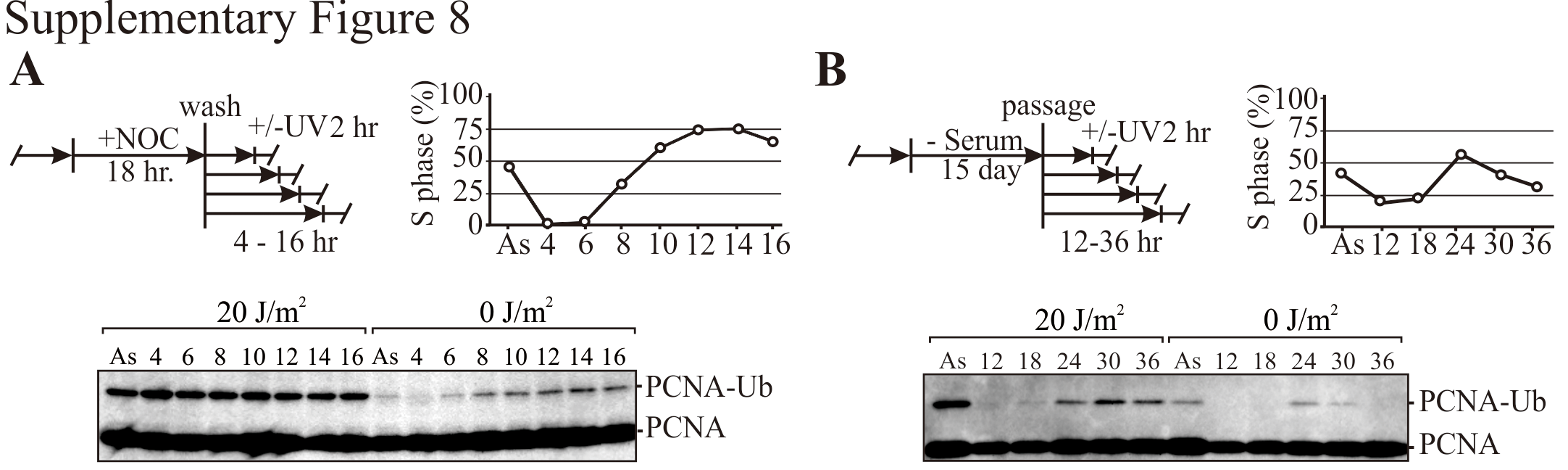

Supplement: S8 Fig — (A) PCNA ubiquitylation during cell cycle progression in human U2OS cells. Top-left, experimental scheme: cells were synchronised with nocodozol, released into fresh media and samples either irradiated, or not irradiated before harvesting at the indicated time points. The fraction of S phase cells was determined by EdU staining (top-right) and PCNA ubiquitylation status was monitored by western blot (bottom). (B) Equivalent experiment using 1BR3hTERT immortalized human fibroblasts synchronised by serum starvation. (TIF) [file pgen.1006789.s008.tif]

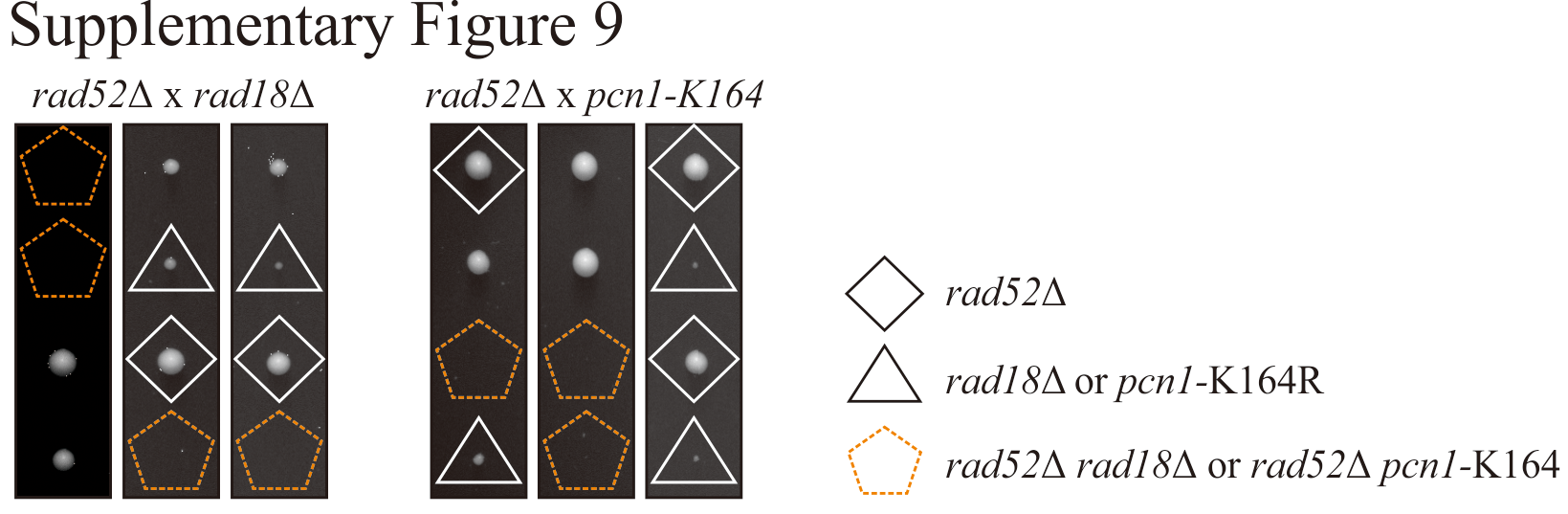

Supplement: S9 Fig — Tetrad analysis of a cross between rad52Δ cells and rad18Δ or pcn1-K164R cells. rad52Δ colonies exhibit a slow growth phenotype whereas rad52Δ rad18Δ or rad52Δ pcn1-K164R double mutants are lethal. (TIF) [file pgen.1006789.s009.tif]

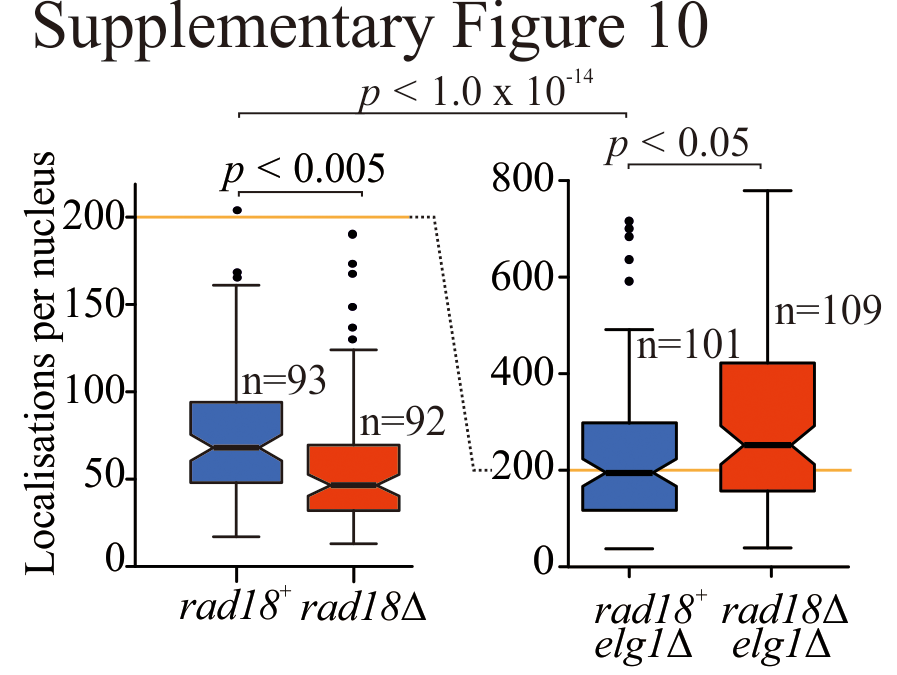

Supplement: S10 Fig — Motion blur of mEos3-PCNA in S phase cells in rad18+ and rad18Δ cells (see Fig 2F) and in the rad18+ elg1Δ and rad18Δ elg1Δ backgrounds. (TIF) [file pgen.1006789.s010.tif]
